# Supplementary material for: Maternal obesity increases the risk of hepatocellular carcinoma through the transmission of an altered gut microbiome
Source: JHEP Rep. 2024 Mar 12;6(5):101056. doi: 10.1016/j.jhepr.2024.101056 (PMC11046215; doi:10.1016/j.jhepr.2024.101056)
Supplement: Multimedia component 1 [file mmc1.pdf]

Maternal obesity increases the risk of hepatocellular carcinoma through the transmission of an altered gut microbiome

Beat Moeckli, Vaihere Delaune, Benoît Gilbert, Andrea Peloso, Graziano Oldani, Sofia El Hajji, Florence Slits, Joana Rodrigues Ribeiro, Ruben Mercier, Adrien Gleyzolle, Laura Rubbia-Brandt, Quentin Gex, Stephanie Lacotte, Christian Toso

Table of contents

Supplementary materials and methods 2

Fig. S1 6

Fig. S2 8

Fig. S3 9

Fig. S4 11

Supplementary references.. 12

Supplementary materials and methods

Transgenic HCC mouse model

LAP-tTA (LAP, B6.NMRI-Tg(Cebpb-tTA)5Bjd/Cnbc, EM:04498) x TRE MYC (B6.Cg-Tg(tetO-MYC,-OVAL)#Gtgm/Ieg, EM:04319) (LAP-Myc) mice were used as HCC transgenic mouse model. Single transgenic LAP-tTA mothers received a HFD for twelve weeks before mating with lean TRE MYC males. Myc expression in the liver was activated by removing the doxycycline enriched diet (Safe Diet, 0.625g/Kg Doxycycline Hyclate) [1,2]. Offspring were assigned to one of the following groups: Stop of doxycycline at 5 weeks with feeding of normal diet (ND) (Dox5w_F1ND), stop of doxycycline at 5 weeks with feeding of choline-deficient methionine controlled high-fat diet (MCD) (Dox5w_F1MCD), or stop of doxycycline at 3 weeks of age with feeding of a ND (Dox3w_F1ND). All animals were sacrificed at 16 weeks.

Physiological and metabolic phenotyping

Body composition (fat and fat-free mass) was determined in vivo using quantitative magnetic resonance (EchoMRI™ 3-in-1 v2.1; Echo Medical Systems, Houston, TX) at different time points. Glycemia was determined using an Accu-Check glucometer (Roche Diabetes Care, Rotkreuz, Switzerland). For fasting glycemia, mice were fasted for 6 hours.

Plasma Analysis

Serum alanine aminotransferase (ALT) and aspartate aminotransferase (AST) levels were analyzed using the cobas c111 clinical chemistry analyzer (Roche Diagnostic, Rotkreuz, Switzerland).

Liver histopathological analysis and immuno-labelling

Liver samples were processed routinely in 10% neutral buffered formalin for 24 h, and then embedded in paraffin. The fixed liver tissues sliced into 5-μm thickness and

stained with haematoxylin and eosin (H&E) and Masson Trichrome (MT) to evaluate liver histology, tumor histology and grading of liver injury. Leukocytes, Macrophages and CD8 T cells were labelled with an anti-CD45 (clone D3F8Q), an anti-Iba1 (clone EPR16588) or an anti-CD8 (clone D4W2Z) followed by an Alexa488-goat anti-rabbit IgG (A32731, Invitrogen) or the SignalStain® Boost IHC Detection Reagent (HRP anti-rabbit, Cell Signaling Technology).

Liver cells apoptosis and proliferation were assessed with anti-cleaved caspase-3 (clone 5A1E) and anti-Ki-67 (clone D3B5) labelling, followed by the SignalStain® Boost IHC Detection Reagent (HRP anti-rabbit, Cell Signaling Technology). Intestinal inflammation and integrity were assessed with anti-Lyzozyme (clone EPR2994) [3,4], anti-Lyve-1 (goat polyclonal AF2125, R&D Systems) [5] or anti-PLVAP (clone E4U6V) labelling [6], followed by the SignalStain® Boost IHC Detection Reagent (HRP anti-rabbit) or by biotin rabbit anti-goat (E0466, Agilent Dako) and HRP-Streptavidin (P0397, Agilent Dako). Labelling was revealed following incubation with 3,3'-diaminobenzidine tetrahydrochloride (DAB, Pierce) and counterstained with haematoxylin.

Assessment and Quantification of steatosis, fibrosis and immuno-labelling

Steatosis, fibrosis and immune-labelling quantifications were performed with the QuPath software package (version 0.3.2 and 0.4.3, QuPath, Edinburgh, UK) [7]. Sagittal sections of the median and the left liver lobe (one each, per animal) and axial sections of intestines (four per animal) were included in the analysis. Whole slide images were scanned with the Zeiss Axioscan-Z1 Microscope Slide Scanner (Carl Zeiss AG, Jena, Germany). Parts of the slide containing tissue were detected in QuPath using the *SimpleTissueDetection* function. The thereby created annotations were automatically assessed with the *createAnnotationFromPixelClassifier* function using a trained pixel classifier to only include liver tissue without large vessels or preparation artefacts.

For steatosis, lipid vacuoles were automatically detected and validated visually in H&E stained slides. The relative area of steatosis was calculated as the area of vacuoles normalized by the total area of the whole slide.

Fibrosis, stained in blue in MT slides was automatically quantified using a manually trained pixel classifier, which discriminates fibrosis based on pixel coloration. By applying this threshold, we determined the percentage of fibrosis on the total surface of each section.

Anti-CD45, anti-Iba-1, anti-CD8, anti-Ki67, anti-cleaved caspase- 3, anti-PLVAP were automatically quantified using an object classifier (Ki67, cleaved caspase-3) or using a pixel classifier (CD45, Iba-1, CD8 and PLVAP) manually trained on 3,3′-Diaminobenzidine (DAB) staining. Hepatocytes and immune cells were discriminated using an object classifier manually trained on nuclei size (hematoxylin staining).

Assessment of intestinal histopathology

The count of Paneth cells was visually conducted on 50 villi per section. Only cells with a clearly visible nucleus and positive staining (lysozyme) throughout the cytoplasm were considered. By dividing the number of counted Paneth cells by the number of vili, the Paneth cell/villus ratio was obtained. This was performed on three sections per specimen. The count of lymphatic vessels (Lyve-1 labelling) was also visually conducted around the entire circumference of the sections. Only lymphatic vessels with a visible lumen and present in the submucosa were counted. By dividing the number of lymphatic vessels by the total number of villi, the lymphatic vessel/villus ratio was obtained. This was performed on three sections per specimen.

Hepatic gene expression

After total RNA extraction of liver tissue (ReliaPrepTM RNA Tissue, Promega, Madison WI, USA), cDNA was synthesised by extending a mix of random primers with the High Capacity cDNA Reverse Transcription Kit in the presence of RNase inhibitor (Applied Biosystems). The relative quantity of each transcript was normalized to the expression of *Eef1*, *Hprt*, and *Gapdh*. SYBRGreen reagent was used for Real-time PCR on the ABI Prism 7000 sequence detection system (Applied

Biosystems, Waltham MA, United States) according to the manufacturer's instructions. Primer sequences are provided in the Supplementary CTAT Table.

Reconstruction of 3D tumor volume

3D reconstructions were performed with liver and bone segmentation via the Imalytics software (Imalytics Preclinical 3.0 (Gremse-IT GmbH, Aachen, Germany). 3D rendering was performed after tumor morphological identification by manually circling ROI in 1mm thick, three axial slice views.

Supplementary data

Fig. S1

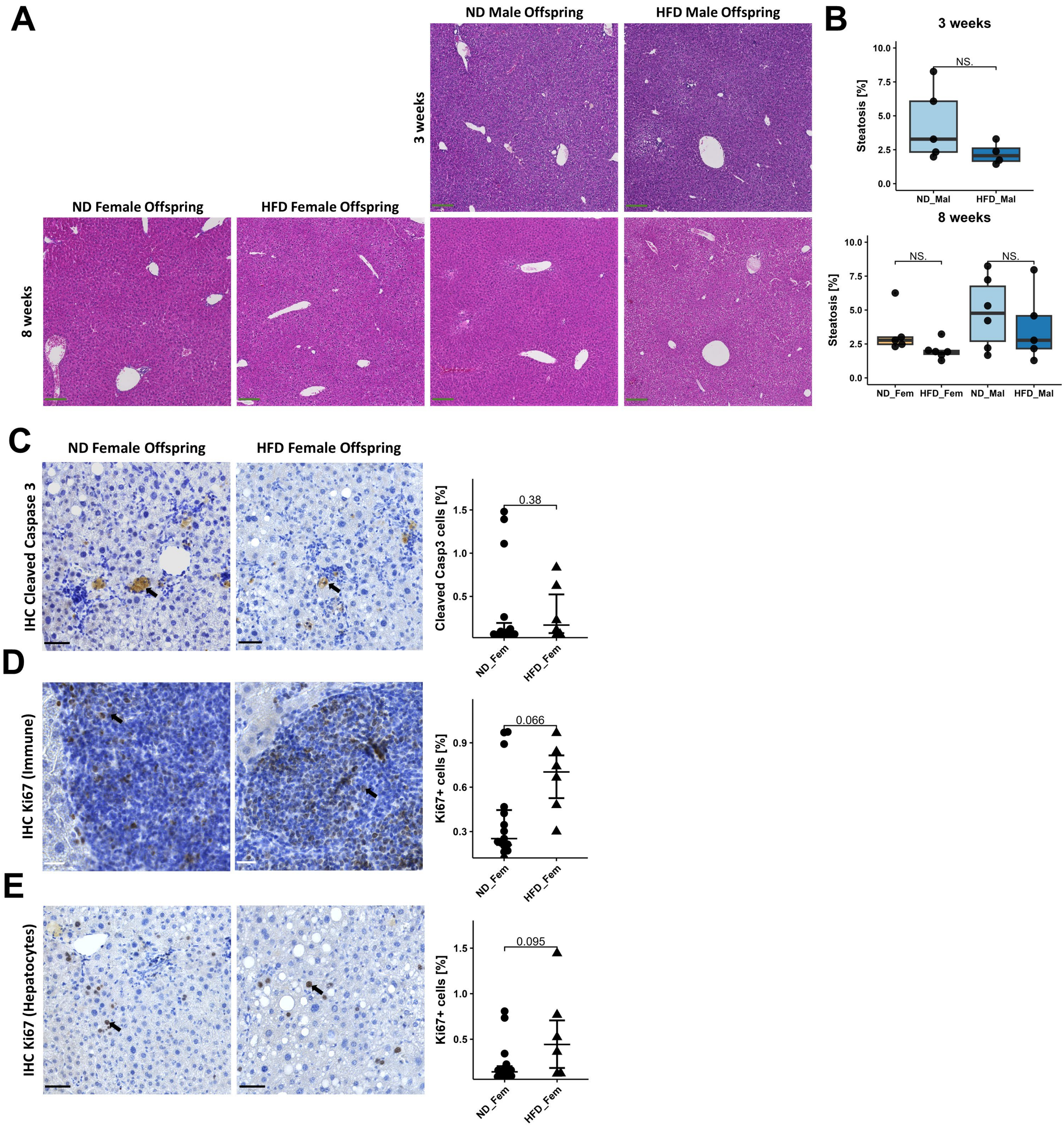

Fig. S1. Maternal obesity does not affect steatosis before adulthood and increases compensatory proliferation Female and male offspring of obese (HFD_Fem & HFD_Mal) and lean mothers (ND_Fem & ND_Mal) were fed a normal diet. (**A**) Histological hematoxylin-eosin stained sections from male offspring at 3 weeks of age, as well as from female and male offspring at 8 weeks of age from ND and HFD mothers. (**B**) Automated quantification of steatosis, expressed as % of liver surface covered by steatotic vesicles. (**C**) Histological sections with immunohistochemistry staining of cleaved caspase 3, (**D**) Ki67 in immune cells, and (**E**) Ki67 in hepatocytes between the two groups with corresponding quantification of stained area. Data presented as median ± IQR, one dot represents one animal, level of significance of p=0.05. Statistical analysis was performed by Wilcoxon–Mann–Whitney test (B, C, D, E). ND_Fem: Female offspring born to lean mothers n = 6-15, F_HFD: Female offspring born to obese mothers n = 6-10, M_ND male offspring born to lean mothers n = 5-7, M_HFD male offspring born to obese mothers n = 5-8. Scale bar Panel A: 100µm, Panels C-E: 50µm.

Fig. S2

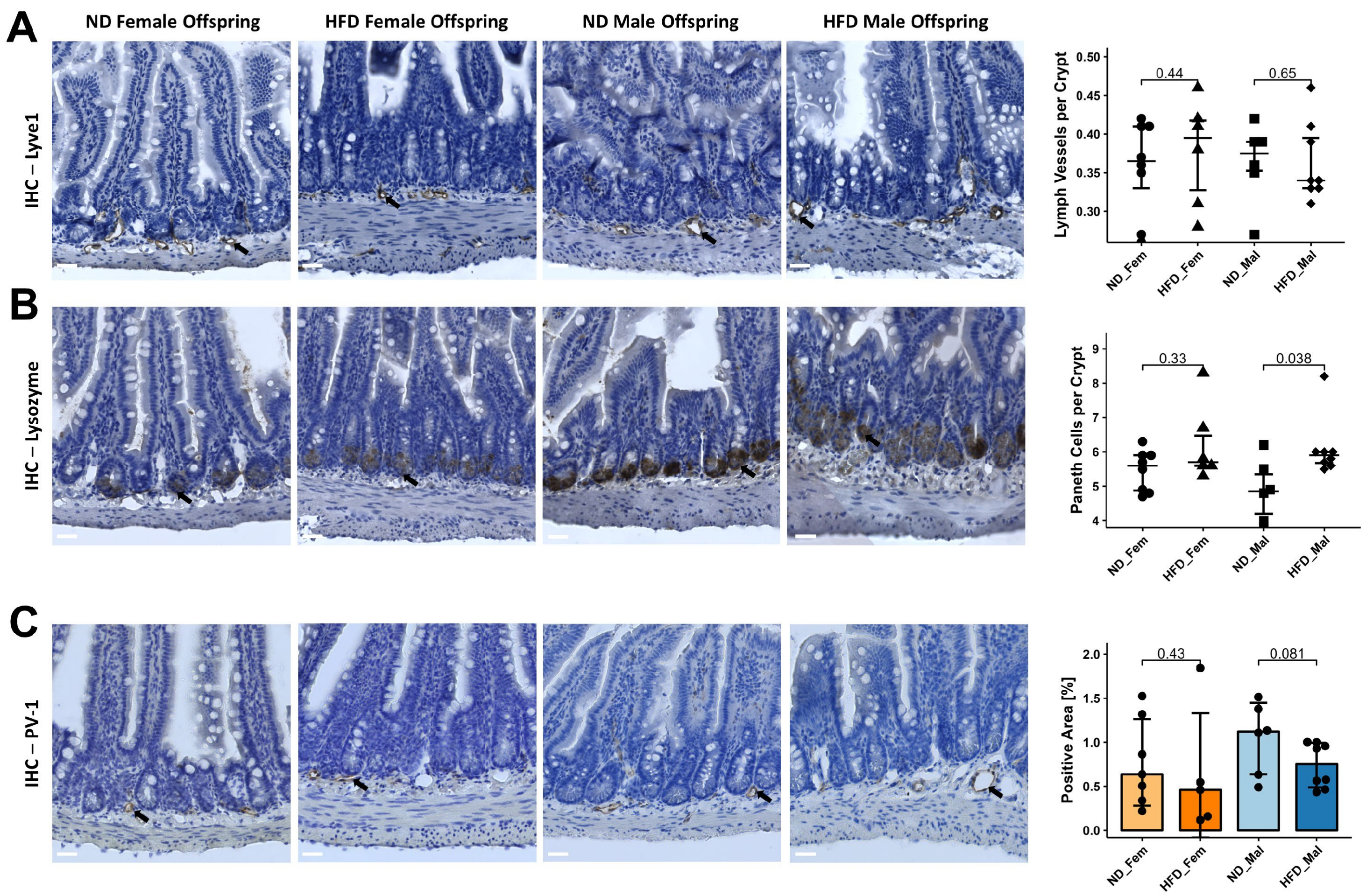

Fig. S2. Maternal obesity does not affect intestinal inflammation and the gut vascular barrier Female and male offspring of obese (HFD_Fem & HFD_Mal) and lean mothers (ND_Fem & ND_Mal) were fed a normal diet. (**A**) Axial histological sections of intestines with Immunohistochemistry staining for lymphatic vessels with anti-LYVE-1. Manual quantification of lymph vessels per crypt (right). (**B**) Axial histological sections of intestines with Immunohistochemistry staining for Paneth cells with anti-lysozyme. Manual quantification of Paneth cells per crypt (right). (**C**) Axial histological sections of intestines immunohistochemistry staining for gut vascular barrier with anti-PV-1. Automated quantification of stained area (right). Data presented as median ± IQR, one dot represents one animal, level of significance of p=0.05. Statistical analysis was performed by Wilcoxon–Mann–Whitney test (A, B, C). ND_Fem: Female offspring born to lean mothers n = 8, F_HFD: Female offspring born to obese mothers n = 7, M_ND male offspring born to lean mothers n = 7, M_HFD male offspring born to obese mothers n = 9. Scale bar: 100µm.

Supplementary data

Fig. S3

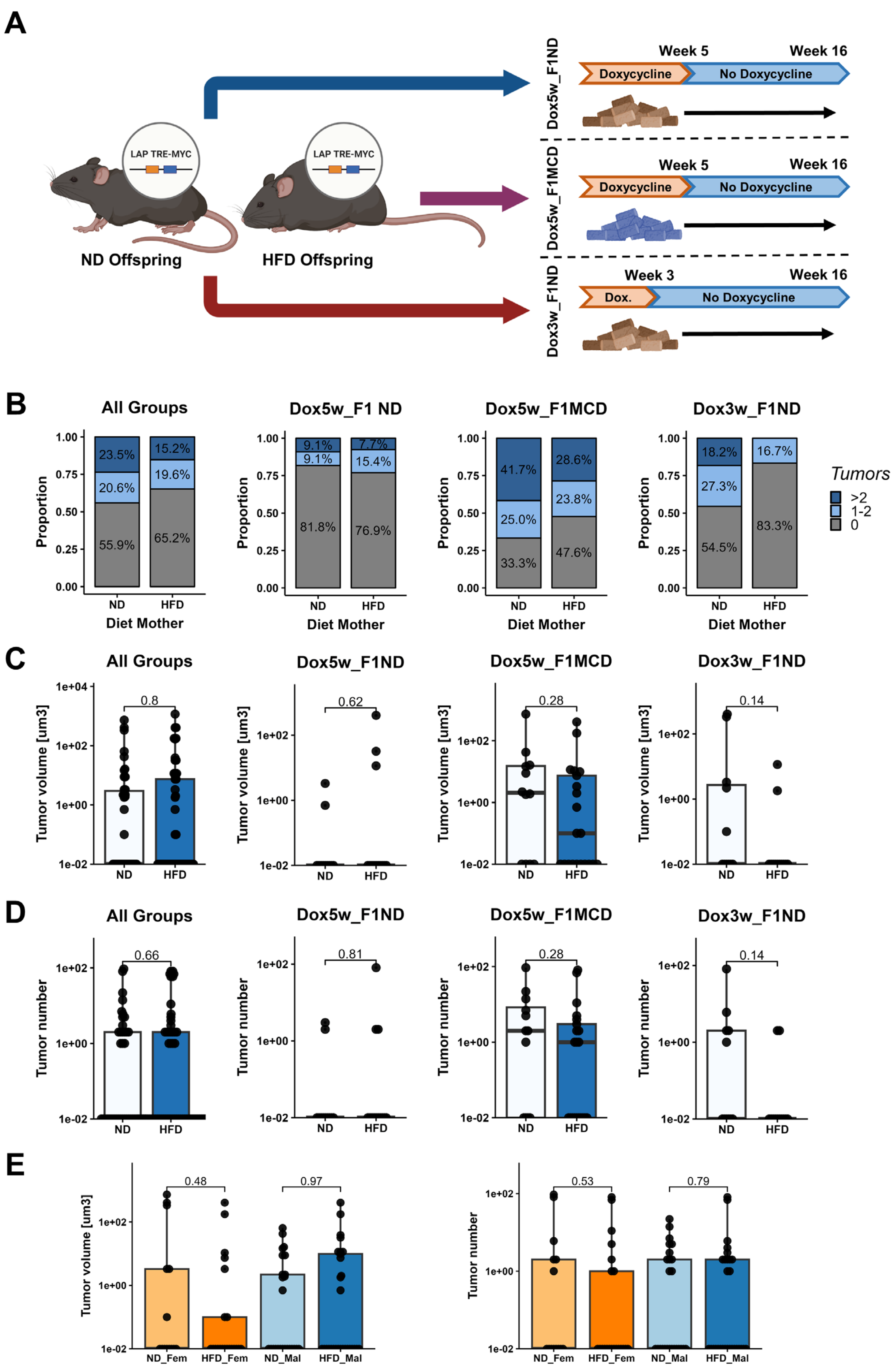

Fig. S3. Maternal obesity does not influence the risk to develop HCC in a short-term transgenic HCC model (**A**) HCC carcinogenesis in double transgenic offspring (LAP-tTA x TRE-MYC) of obese or lean mothers was induced through removal of doxycycline. Offspring were assigned to one of the following groups: Stop of doxycycline at 5 weeks with feeding of normal diet (ND) (Dox5w_F1ND), stop of doxycycline at 5 weeks with feeding of methionine choline deficient diet (MCD) (Dox5w_F1MCD), or stop of doxycycline at 3 weeks of age with feeding of a ND (Dox3w_F1ND) All animals were sacrificed at 16 weeks. (**B**) The proportion of animals that developed 0, 1-2 or more than 2 tumors for all groups on the left or per group on the right. (**C**) The total number of tumors per animal and (D) the total tumor volume per animal. (**E**) The total tumor volume or number differentiated by diet of the mother and sex of the offspring. Data presented as median ± IQR, one dot represents one animal, level of significance of p=0.05. Statistical analysis was performed by Wilcoxon–Mann–Whitney test (C, D, E). Median with interquartile ranges. Dox5w_F1ND n=23 (ND=11 and HFD=12), Dox5w_F1MCD n=33 (ND=12, HFD=21, Dox3w_F1ND n=24 (ND=11, HFD=13).

Fig. S4

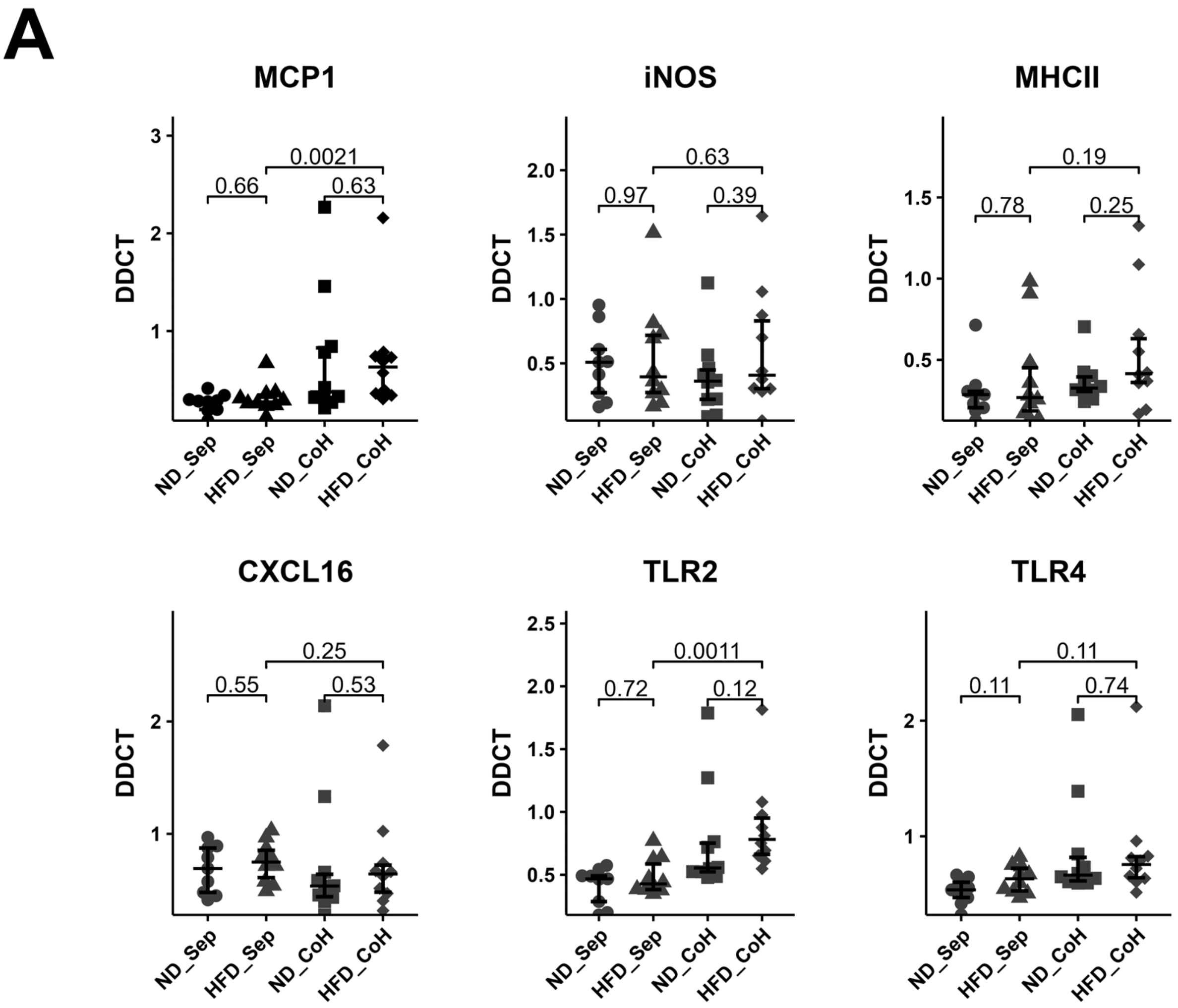

Fig. S4. Expression of inflammatory markers or Toll-like receptor family members in DEN treated offspring Offspring of obese and lean mothers were either separately housed (ND_Sep and HFD_Sep) or co-housed (ND_CoH and HFD_CoH) after weaning. (**A**) Real-time PCR expression analysis of inflammatory markers such as Monocyte Chemoattractant Protein-1 (MCP-1), inducible Nitric Oxide Synthase (iNOS), Class II Major Histocompatibility Complex (MHC II) molecules, and chemokine CXCL16. Expression of Toll-like Receptor (TLR) family member 2 and 4. Data presented as median ± IQR, one dot represents one animal, level of significance of p=0.05. Statistical analysis was performed by Wilcoxon–Mann–Whitney test. ND_Sep: Female offspring born to lean mothers housed seperately n = 10, HFD_Sep: Female offspring born to obese mothers housed separately n = 10, ND_CoH female offspring born to lean mothers co-housed with offspring of obese mothers n = 10, HFD_CoH female offspring born to obese mothers cohoused with offspring of lean mothers n = 10

Supplementary references

[1] Felsher DW, Bishop JM. Reversible Tumorigenesis by MYC in Hematopoietic Lineages. Mol Cell 1999;4:199–207. https://doi.org/10.1016/S1097-2765(00)80367-6.

[2] Shachaf CM, Kopelman AM, Arvanitis C, Karlsson Å, Beer S, Mandl S, et al. MYC inactivation uncovers pluripotent differentiation and tumour dormancy in hepatocellular cancer. Nature 2004;431:1112–7. https://doi.org/10.1038/nature03043.

[3] Jackson DN, Panopoulos M, Neumann WL, Turner K, Cantarel BL, Thompson-Snipes L, et al. Mitochondrial dysfunction during loss of prohibitin 1 triggers Paneth cell defects and ileitis. Gut 2020;69:1928–38. https://doi.org/10.1136/gutjnl-2019-319523.

[4] Clevers HC, Bevins CL. Paneth cells: maestros of the small intestinal crypts. Annu Rev Physiol 2013;75:289–311. https://doi.org/10.1146/annurev-physiol-030212-183744.

[5] Johnson LA, Banerji S, Lawrance W, Gileadi U, Prota G, Holder KA, et al. Dendritic cells enter lymph vessels by hyaluronan-mediated docking to the endothelial receptor LYVE-1. Nat Immunol 2017;18:762–70. https://doi.org/10.1038/ni.3750.

[6] Mouries J, Brescia P, Silvestri A, Spadoni I, Sorribas M, Wiest R, et al. Microbiota-driven gut vascular barrier disruption is a prerequisite for non-alcoholic steatohepatitis development. J Hepatol 2019;71:1216–28. https://doi.org/10.1016/j.jhep.2019.08.005.

[7] Bankhead P, Loughrey MB, Fernández JA, Dombrowski Y, McArt DG, Dunne PD, et al. QuPath: Open source software for digital pathology image analysis. Sci Rep 2017;7:16878. https://doi.org/10.1038/s41598-017-17204-5.
