# Supplementary material for: Maternal obesity increases the risk of hepatocellular carcinoma through the transmission of an altered gut microbiome
Source: JHEP Rep. 2024 Mar 12;6(5):101056. doi: 10.1016/j.jhepr.2024.101056 (PMC11046215; doi:10.1016/j.jhepr.2024.101056)
Supplement: Multimedia component 2 [file mmc2.docx]

**Journal of Hepatology**

**CTAT methods**

- 1. **Antibodies**

| **Name** | **Citation** | **Supplier** | **Cat no.** | **Clone no.** |
| --- | --- | --- | --- | --- |
| CD45 |  | Cell Signaling Technology | #70257 | D3F8Q |
| Iba-1 |  | Abcam | ab178846 | EPR16588 |
| CD8a |  | Cell Signaling Technology | #98941 | D4W2Z |
| Cleaved caspase-3 |  | Cell Signaling Technology | #9664 | 5A1E |
| Ki-67 |  | Cell Signaling Technology | #9129 | D3B5 |
| PLVAP |  | Cell Signaling Technology | #24764 | E4U6V |
| Lyzozyme |  | Abcam | ab108508 | EPR2994 |
| Lyve-1 |  | R&D Systems | AF2125 | Polyclonal |
| SignalStain® Boost IHC Detection Reagent |  | Cell Signaling Technology | #8114 | Polyclonal |
| biotin rabbit anti-goat |  | Agilent Dako | E0466 | Polyclonal |
| HRP-streptavidin |  | Agilent Dako | P0397 | Polyclonal |
| Alexa488-goat anti-rabbit IgG |  | Invitrogen | A32731 | polyclonal |

- 1. **Organisms**

| **Name** | **Citation** | **Supplier** | **Strain** | **Sex** | **Age** | **Overall n number** |
| --- | --- | --- | --- | --- | --- | --- |
| C57BL/6N |  | Charles River Laboratories | C57BL/6N | Male and female | 4 to 40weeks | 200 |
| (LAP-Myc x)F1 | DOI: [10.1002/hep.22652](https://doi.org/10.1002/hep.22652) | Infrafrontier | (EM:04319  X EM:04498) F1 | Male and female | 16 weeks | 80 |

- 1. **Sequence based reagents**

| **Name** | **Sequence** | **Supplier** |
| --- | --- | --- |
| m-TGFb-F | GTCCTTGCCCTCTACAACCA | Microsynth AG |
| m-TGFb-R | GTTGGACAACTGCTCCACCT | Microsynth AG |
| m-Col1a1-F | CCTGAGTCAGCAGATTGAGAACA | Microsynth AG |
| m-Col1a1-R | CCAGTACTCTCCGCTCTTCCA | Microsynth AG |
| m-Timp1 | GATATGTCCACAAGTCCCAGAACC | Microsynth AG |
| m-Timp1-R | CCACAGCCAGCACTATAGGTCTTT | Microsynth AG |
| m-Vimentin-F | CGGAAAGTGGAATCCTTGCAGG | Microsynth AG |
| m-Vimentin-R | AGCAGTGAGGTCAGGCTTGGAA | Microsynth AG |
| m-tlr2-F | ACAGCAAGGTCTTCCTGGTTCC | Microsynth AG |
| m-tlr2-R | GCTCCCTTACAGGCTGAGTTCT | Microsynth AG |
| m-tlr4-F | GGCAGCAGGTGGAATTGTAT | Microsynth AG |
| m-tlr4-R | AGGATTCGAGGCTTTTCCAT | Microsynth AG |
| m-MHCII-F | ACTGCCATTACCTGTGCCTTAGAG | Microsynth AG |
| m-MHCII-R | CCATGAACTGGTACACGAAATGCC | Microsynth AG |
| m-iNOS-F | AAT CTT GGA GCG AGT TGT GG | Microsynth AG |
| m-iNOS-R | CAG GAA GTA GGT GAG GGC TTG | Microsynth AG |
| mMCP-1-F | GCT GAC CCC AAG AAG GAA TG | Microsynth AG |
| mMCP-1-R | GCT GAA GAC CTT AGG GCA GA | Microsynth AG |
| m-CXCL16-F | CATTCTTCGGGAACCACAGCTTG | Microsynth AG |
| m-CXCL16-R | GGTCAGCCAAGACTATCCGACAAA | Microsynth AG |
| m-EEF1-F | TCCACTTGGTCGCTTTGCT | Microsynth AG |
| m-EEF1-R | CTTCTTGTCCACAGCTTTGATGA | Microsynth AG |
| m-GAPDH-F | TCCATGACAACTTTGGCATTG | Microsynth AG |
| m-GAPDH-R | CAGTCTTCTGGGTGGCAGTGA | Microsynth AG |
| m-HPRT-F | GCTCGAGATGTCATGAAGGAGAT | Microsynth AG |
| m-HPRT-R | AAAGAACTTATAGCCCCCCTTGA | Microsynth AG |

- 1. **Deposited data**

| **Name of repository** | **Identifier** | **Link** |
| --- | --- | --- |
| Yareta | 10.26037/yareta:ef2h6hlu5zc3zhw6fiuywsj54m | <https://doi.org/10.26037/yareta:ef2h6hlu5zc3zhw6fiuywsj54m> |

- 1. **Software**

| **Software name** | **Manufacturer** | **Version** |
| --- | --- | --- |
| QuPath | <https://qupath.github.io>. | 0.3.2 and 0.4.3 |
| R | GNU Project | 4.0.3 et 4.1.2 |
| R Studio | GNU Project | 2022.02.3 |
| OsiriX DICOM viewer software | Pixmeo SARL | 12.0 |
| Imalytics | Gremse-IT | 3.0 |

- 1. **Other (diet)**

| ND (diet) | Envigo TD.120455 |
| --- | --- |
| HFD (diet) | Envigo TD.08811 |
| N-nitrosodiethylamine (DEN) | Sigma-Aldrich N0756 |
| MCD (diet) | Safe Diet U8958 Version 347 |

- 1. **Please provide the details of the corresponding methods author for the manuscript:**

| PD Dr. med. Stéphanie Lacotte  Transplantation and Hepatology Laboratory  Centre Medical Universitaire, C01.1530a  Rue Michel Servet, 1  1206 Geneva, Switzerland  E-Mail: stephanie.lacotte@unige.ch  Phone: +41 22 379 56 61 |
| --- |
