# Supplementary material for: Maternal obesity increases the risk of hepatocellular carcinoma through the transmission of an altered gut microbiome
Source: JHEP Rep. 2024 Mar 12;6(5):101056. doi: 10.1016/j.jhepr.2024.101056 (PMC11046215; doi:10.1016/j.jhepr.2024.101056)
Supplement: Multimedia component 4 [file mmc4.pdf]

# Maternal obesity increases the risk of hepatocellular carcinoma through the transmission of an altered gut microbiome

Beat Moeckli,[1,2,*] Vaihere Delaune,[1,2] Benoît Gilbert,[3,4] Andrea Peloso,[2] Graziano Oldani,[1,5] Sofia El Hajji,[1,2] Florence Slits,[1] Joana Rodrigues Ribeiro,[2] Ruben Mercier,[1] Adrien Gleyzolle,[6] Laura Rubbia-Brandt,[7] Quentin Gex,[1] Stephanie Lacotte,[1] Christian Toso[1,2,*]

*[1]Hepatology and Transplantation Laboratory, Department of Surgery, Faculty of Medicine, University of Geneva, 1206 Geneva, Switzerland; [2]Department of Surgery, Division of Visceral Surgery, Geneva University Hospitals, 1205 Geneva, Switzerland; [3]Department of Medicine, Division of Rheumatology, Geneva University Hospitals, 1206 Geneva, Switzerland; [4]Geneva Centre for Inflammation Research (GCIR), Faculty of Medicine, University of Geneva, Geneva, Switzerland; [5]Department of Surgery, Division of General Surgery, The University of British Columbia, Vancouver, Canada; [6]Department of Diagnostics, Division of Radiology, Geneva University Hospitals, 1205 Geneva, Switzerland; [7]Department of Diagnostics Division of Clinical Pathology, Geneva University Hospitals, 1205 Geneva, Switzerland*

JHEP Reports **2024.** https://doi.org/10.1016/j.jhepr.2024.101056

**Background & Aims:** Emerging evidence suggests that maternal obesity negatively impacts the health of offspring. Additionally, obesity is a risk factor for hepatocellular carcinoma (HCC). Our study aims to investigate the impact of maternal obesity on the risk for HCC development in offspring and elucidate the underlying transmission mechanisms.
**Methods:** Female mice were fed either a high-fat diet (HFD) or a normal diet (ND). All offspring received a ND after weaning. We studied liver histology and tumor load in a N-diethylnitrosamine (DEN)-induced HCC mouse model.
**Results:** Maternal obesity induced a distinguishable shift in gut microbial composition. At 40 weeks, female offspring of HFD-fed mothers (HFD offspring) were more likely to develop steatosis (9.43% *vs.* 3.09%, $p = 0.0023$) and fibrosis (3.75% *vs.* 2.70%, $p = 0.039$), as well as exhibiting an increased number of inflammatory infiltrates (4.8 *vs.* 1.0, $p = 0.018$) and higher expression of genes involved in fibrosis and inflammation, compared to offspring of ND-fed mothers (ND offspring). A higher proportion of HFD offspring developed liver tumors after DEN induction (79.8% *vs.* 37.5%, $p = 0.0084$) with a higher mean tumor volume (234 *vs.* 3 $\mu m^3$, $p = 0.0041$). HFD offspring had a significantly less diverse microbiota than ND offspring (Shannon index 2.56 *vs.* 2.92, $p = 0.0089$), which was rescued through co-housing. In the principal component analysis, the microbiota profile of co-housed animals clustered together, regardless of maternal diet. Co-housing of HFD offspring with ND offspring normalized their tumor load.
**Conclusions:** Maternal obesity increases female offspring's susceptibility to HCC. The transmission of an altered gut microbiome plays an important role in this predisposition.
**Impact and implications:** The worldwide incidence of obesity is constantly rising, with more and more children born to obese mothers. In this study, we investigate the impact of maternal diet on gut microbiome composition and its role in liver cancer development in offspring. We found that mice born to mothers with a high-fat diet inherited a less diverse gut microbiome, presented chronic liver injury and an increased risk of developing liver cancer. Co-housing offspring from normal diet- and high-fat diet-fed mothers restored the gut microbiome and, remarkably, normalized the risk of developing liver cancer. The implementation of microbial screening and restoration of microbial diversity holds promise in helping to identify and treat individuals at risk to prevent harm for future generations.
© 2024 The Authors. Published by Elsevier B.V. on behalf of European Association for the Study of the Liver (EASL). This is an open access article under the CC BY license (http://creativecommons.org/licenses/by/4.0/).

## Introduction

The rising incidence of obesity worldwide is emerging as a major public health issue.[1] Of particular worry is the situation faced by women of reproductive age: over 60% of women aged 20 to 39 years in the United States are either overweight or obese, with this trend showing a concerning increase.[2] Obesity is associated with a spectrum of chronic liver diseases and can lead to metabolic-associated steatotic liver disease (MASLD) in 75% of patients and metabolic-associated steatohepatitis in 34% of patients.[3] MASLD is defined as the presence of hepatic steatosis with the finding of a cardiometabolic risk factor and no other cause of hepatic steatosis.[4] Importantly, one third of patients with MASLD can progress to cirrhosis over time and eventually develop hepatocellular carcinoma (HCC).[5] When compared to the general population, MASLD is associated with a 17-fold

Keywords: Maternal obesity; hepatocellular carcinoma; cancer; overweight; obesity; gut microbiota; microbiome; gut vascular barrier.
*Received 14 July 2023; received in revised form 13 February 2024; accepted 19 February 2024; available online 12 March 2024*
* Corresponding authors. Addresses: Division of Digestive Surgery, Department of Surgery, University Hospitals of Geneva, Rue Gabrielle-Perret-Gentil 4, 1205 Geneva, Switzerland; Tel.: +41 22 379 56 61. (C. Toso) or Transplantation & Hepatology Lab, Department of Surgery, University of Geneva, Rue Michel-Servet 1, 1205 Geneva, Switzerland; +41 22 379 56 54. (B. Moeckli).
*E-mail addresses:* beat.moeckli@unige.ch (B. Moeckli), christian.toso@hcuge.ch (C. Toso).

ELSEVIER

excess risk for HCC.[6,7] HCC is the third leading cause of cancer mortality worldwide with a 5-year survival rate of below 20%.[8]

In addition to its direct impact on the health of mothers, excess bodyweight also impairs the long-term health of offspring. Clinical studies have shown that maternal overweight/obesity increases the risk of metabolic disease and obesity, and impairs neurocognitive development, in offspring.[9–12] Murine studies have revealed that maternal obesity promotes MASLD and inflammatory liver changes in offspring.[13–16] A recent clinical study confirmed that maternal obesity is strongly associated with the risk for MASLD in young adults.[17] In addition, recent large-scale prospective cohort studies have shown that descendants of obese mothers demonstrate an increased risk for childhood cancers and colorectal cancer during adulthood.[18–20]

With regards to liver cancer, Sun *et al.* studied the effect of a maternal high-fat diet (HFD) on the risk of developing HCC in a N-diethylnitrosamine (DEN)-induced murine liver cancer model.[21] The authors observed an increased tumor load in the offspring of mothers fed a HFD. The latter was associated with inflammatory changes in the peri-tumoral liver tissue and increased tumor load in subsequent generations of offspring. The study linked this increased tumor load to a gradual down-regulation of two metabolic genes, Acyl-CoA synthetase long chain family member 1 *(Acsl1)* and aldehyde dehydrogenase family member 2 *(Aldh2)* mediated through a specific miRNA, miR-27a-3p. Sun *et al.* thus provided compelling evidence on the impact of maternal obesity on liver cancer risk in offspring. However, in a recent analysis of transcriptomic data from ten different studies, we found no differential expression of *Acsl1* and *Aldh2* in offspring of obese mothers.[22] Therefore, other mechanisms besides the regulation of specific metabolic genes are likely implicated.

Obesity alters the composition of the microbiome and this dysbiosis is transmitted at the time of birth from the mother to her offspring.[23] Germ-free mice colonized with stool microbes from infants born to obese mothers develop periportal inflammation and higher rates of MASLD when fed a Western-style diet.[24] However, the role of the gut microbiome in the risk transmission for cancer from mother to offspring remains poorly understood.

The aim of this study is to evaluate the impact of maternal obesity on the risk of developing HCC in murine offspring and gain insight into the mechanisms involved, with a specific focus on the gut microbiome.

## Materials and methods

Further details regarding the materials and methods used are provided in the supplementary materials and methods.

### Animals and housing

Animal experimentation was carried out under the terms of an experimental protocol approved by the ethical committee of the University of Geneva and the Geneva veterinary authorities (GE56-20 and GE85). All mice were housed in the animal facility of the University of Geneva under 12/12 h light/dark cycles with free access to food and water. Dams (C57BL/6N) were purchased from Charles River Laboratories (Ecully, France). Dams were randomly assigned to either a normal diet (ND: 17% kcal fat, 61% kcal carbohydrate, 22% kcal protein; Envigo TD.120455) or an energy-rich Western-style HFD (45% kcal fat, 41% kcal carbohydrate, 15% kcal protein; Envigo TD.08811). For chemical induction of HCC, offspring were injected with DEN (Sigma-Aldrich) at 25 mg/kg body weight intra-peritoneally at 14 days of age. After weaning, all offspring were fed a normal diet (ND) until euthanasia at 40 weeks.

To normalize the gut microbiome, offspring of HFD-fed mothers (hereafter HFD offspring) were co-housed together with offspring of ND-fed mothers (hereafter ND offspring) at a ratio of 1:1 after weaning at 21 days. Tumors were induced through DEN induction at 14 days of age as mentioned above.

### Sample collection and microbial 16S rRNA gene extraction

Fecal samples were collected under aseptic conditions from offspring at week 12 and at euthanasia. Stool samples were snap-frozen in liquid nitrogen and bulk-shipped to Microbiome Insights (Vancouver, BC, Canada) for DNA extraction and 16S rRNA sequencing and analysis. 16Sv4 amplicons generated from stool pellet samples were sequenced on a MiSeq platform. MiSeq-generated Fastq files were used for bioinformatics analyses.

### 16S rRNA gene sequence filtering, denoising, chimera removal, and taxonomic assignment

Fastq files were filtered and trimmed using the DADA2 pipeline (v1.16.0) on R (v4.0.3).[25,26] Reads were truncated after 230 (forward) and 150 (reverse) nucleotides. Denoising, merging and chimera removal were performed with default parameters. This generated a set of amplicon sequence variants (ASV), which were subsequently matched to the Silva 16S database (v138) using the DADA2 built-in assigner.[27]

### 16S-data analysis and figure generation

The output of the DADA2 pipeline was visualized on R with packages *phyloseq* (v1.32.0) and *ggplot2* (v3.3.6).[25,28,29] Sample richness was assessed using the Shannon index. For principal component analysis (PCA), ASV counts were transformed into proportions, aggregated at the relevant taxonomic level (*i.e.* family level), and used as input for PCA before visualization using ggplot2. For principal coordinate analysis, ASV counts were transformed into proportions, and samples were ordinated using a Bray-Curtis dissimilarity matrix (at the ASV level), before PCA plotting.

To assess taxa-specific differential expression between two groups, at a certain taxonomical level, low abundance ASV were removed. Then ASV in this filtered dataset were aggregated at the relevant taxonomical level (*i.e.* Family level), and relative proportions were compared between groups using multiple Wilcoxon tests. Finally, the resulting output was plotted, ordering by relative difference in abundance. As a sensitivity analysis, the differential analysis was also performed using package Aldex2, with sequence counts as input. Aldex2 performs a centered-log-ratio transformation on the count data, a recommended method to account for data compositionality.[30]

### *In vivo* micro-CT imaging for tumor quantification

*In vivo* imaging was performed using a micro-CT system (Quantum GX, PerkinElmer, Hopkinton, MA, USA) 24 h after intra-venous injection of a contrast agent (ExiTron nano 12000, 100 μl/animal; Miltenyi Biotec, Bergisch-Gladbach, Germany). All *in vivo* imaging procedures were performed under inhaled isoflurane anesthesia. The analysis of the images was performed using OsiriX DICOM viewer software (version 12.0). Tumor morphological analysis was performed on sets of axial images using a slice thickness of 1 mm and manually contouring each

individual tumor (regions of interest). Tumor volumes were then automatically calculated based on the regions of interest defined by the user. Analysis of tumor volume by micro-CT was performed at 24, 32 and 36 weeks of age.

### Statistical analysis

Descriptive statistics were used to summarize groups with data displayed as median ± interquartile range or where appropriate as mean ± SEM with individual points shown after normality testing. Normality distribution testing was performed using Shapiro-Wilk's test. Groups were compared by non-parametric Wilcoxon signed-rank test, parametric unpaired *t* test, one-way or two-way repeated-measures analysis of variance (ANOVA) with *post hoc* Tukey's multiple comparison test or non-parametric unpaired Wilcoxon or Kruskal-Wallis test with Holm correction for multiple comparisons, as appropriate. All statistical analyses and graphical representations were performed using the R statistical programming environment (R version 4.1.2/RStudio version 2022.02.3). For all analyses, a two-tailed probability of type 1 error of 0.05 was considered significant.

## Results

### Maternal obesity alters the composition of the gut microbiota in offspring

We assigned female C57BL/6N mice to either a HFD or a ND for 12 weeks before mating with lean males. Dams fed a HFD developed obesity and weighed significantly more than dams fed a ND before the start of breeding (28.3 *vs.* 21.4 g, $p$ <0.001) (Fig. 1B). After birth and during lactation, the offspring remained with the mother and their respective diet (HFD or ND) until weaning at 21 days of age. After weaning, all offspring were fed a ND (Fig. 1A). Body weight of the offspring was measured weekly beginning after weaning on post-natal day 21. Female offspring of obese mothers weighed significantly more immediately after weaning at week 4 (17.7 *vs.* 15.4 g, $p$ <0.001), during adult life at week 20 (29.5 *vs.* 25.0, $p$ = 0.002) but not at sacrifice at week 40 (35.8 *vs.* 35.3, $p$ = 0.91) (Fig. 1B, left). Male offspring of obese mothers weighed significantly more immediately after weaning at week 4 (20.0 *vs.* 18.6 g, $p$ = 0.009), but not during adult life (34.5 *vs.* 32.0 g, $p$ = 0.28) nor at sacrifice (40.7 *vs.* 42.1 g, $p$ = 0.38) (Fig. 1B, right). Female offspring of obese mothers showed a trend towards increased fat mass, measured by EchoMRI at 8 weeks (3.2 *vs.* 2.5 g, $p$ = 0.093), with no statistical difference at 40 weeks; there were no differences in male offspring at any time point (Fig. 1C). With regards to markers of liver injury, female offspring of obese mothers displayed higher serum aspartate aminotransferase (167 *vs.* 66 IU/L, $p$ = 0.0021) and alanine aminotransferase (167 *vs.* 66 IU/L, $p$ = 0.014) levels compared to the female offspring of lean mothers at 40 weeks of age (Fig. 1D), but not before.

To ascertain the lasting effect of maternal obesity on the gut microbiome of offspring, we analyzed bacterial 16S ribosomal RNA genes of fecal samples collected from female HFD and ND offspring at 40 weeks. At the phylum level, female HFD offspring showed an altered gut microbial composition (Fig. 1E, left). At week 40, maternal obesity induced a distinguishable shift in the microbial composition, with a trend towards increased proportions of Actinobacteriota (16.9 *vs.* 6.0%, $p$ = 0.15), Verrucomicrobiota (28.5 *vs.* 23.0%, $p$ = 0.22) and Firmicutes (22.2 *vs.* 17.4%, $p$ = 0.22) in the principal component analysis (Fig. 1E,F). On the other hand, the gut microbiota of female ND offspring was characterized by higher levels of Bacteroidota (52.9 *vs.* 31.4%, $p$ = 0.0079) and Desulfobacterota (0.092 *vs.* 0.0057%, $p$ = 0.011) (Fig. 1E,F). The decreased proportion of Bacteroidota is consistent with published literature on the effect of obesity on the gut microbiome.[31]

### Maternal obesity predisposes offspring to steatosis and fibrosis

To study the impact of maternal obesity and the altered gut microbiome of offspring on the risk of developing chronic liver disease and MASLD, we performed histological sections and quantified steatosis, with a specific focus on the timeline of MASLD development. At weaning and at 8 weeks of age we detected low levels of steatosis in offspring without significant differences between groups (Fig. S1). At 20 weeks of age, low levels of steatosis started to appear with a higher surface of liver tissue covered by steatosis for male HFD offspring (3.74 *vs.* 2.47%, $p$ = 0.021) and a trend towards more steatosis in female HFD offspring (3.01 *vs.* 2.26, $p$ = 0.12) (Fig. 2A,B). At 40 weeks, the rate of steatosis for male and female ND offspring remained constant while female HFD offspring developed significant macro- and micro-vesicular steatosis (9.43 *vs.* 3.09%, $p$ = 0.0016) (Fig. 2A-C). Previous murine studies have shown that maternal obesity increases the risk of steatosis and MASLD.[13,14] However, most studies sacrificed the offspring before week 20 and exacerbated the development of MASLD by feeding offspring a HFD. Our study shows that MASLD only develops later in life but that it can develop in offspring fed a ND.

In addition to steatosis, we also assessed fibrosis in offspring at an adult age. Both female (3.75 *vs.* 2.70%, $p$ = 0.039) and male (3.52 *vs.* 2.20%, $p$ = 0.011) HFD offspring exhibited more fibrosis than their ND counterparts (Fig. 2D,E). In addition to the increased amount of fibrosis, we also found a significant overexpression of several pro-fibrotic markers in the livers of female HFD offspring at 40 weeks (Fig. 2F). In contrast to steatosis, where the observed effect was stronger for females, fibrosis seemed to affect both sexes equally. As an additional marker of liver injury, we also observed increased compensatory proliferation in immune cells and hepatocytes in female HFD offspring at 40 weeks (Fig. S1D,E).

### Maternal obesity increases hepatic inflammation

To study the influence of maternal obesity on hepatic inflammation we analyzed the number and size of immune cell infiltrates in the hepatic tissue of offspring. In line with liver aminotransferase levels (Fig. 1D) and the development of steatosis (Fig. 2), none of the offspring groups exhibited a significant presence of immune cell infiltrates in the liver before or at 20 weeks (Fig. 3A,B).

At 40 weeks of age, female HFD offspring exhibited a significant increase in inflammatory infiltrates covering 28.2% of the liver tissue surface, in contrast to 12.3% for ND offspring ($p$ = 0.011) (Fig. 3A,B). As these results were only of significance in the female offspring population, we then further studied the cellular composition of these inflammatory liver infiltrates in the livers of female offspring at 40 weeks. Female HFD offspring exhibited increased CD45 staining (4.8 *vs.* 3.9%, $p$ = 0.045) (Fig. 3C), no differences in Iba1 staining (8.5 *vs.* 8.2, $p$ = 0.83) (Fig. 3D), and increased CD8 staining (0.74 *vs.* 0.30%, $p$ = 0.048) (Fig. 3E).

To confirm these histological findings, we studied the expression of inflammatory markers and Toll-like receptors (TLRs) in the liver tissue in female offspring at 40 weeks. TLRs are

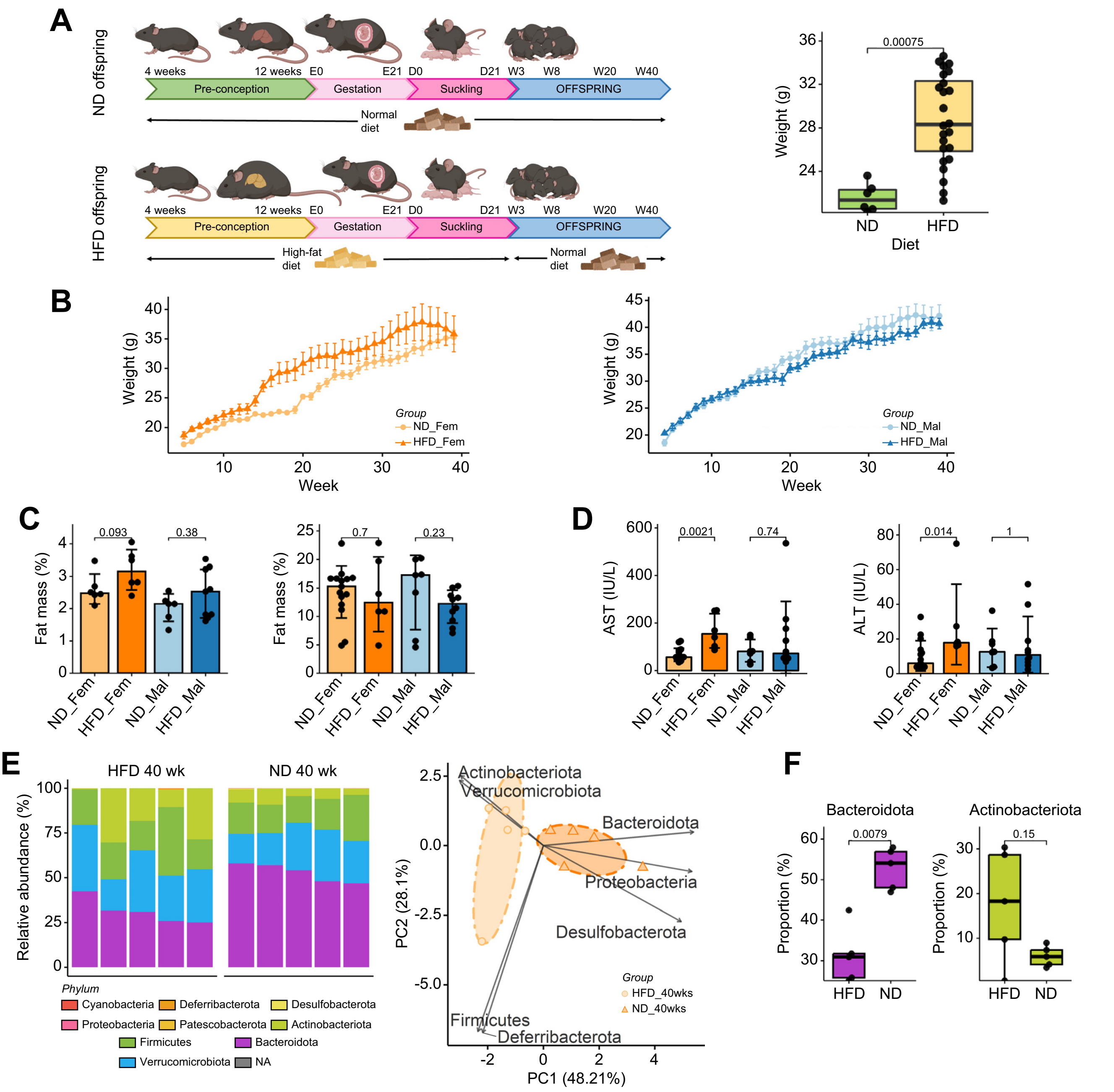


**Fig. 1. Maternal obesity alters the composition of the gut microbiota in offspring** (A) Experimental flowchart, female mice were fed either a HFD or a ND for 12 weeks before mating, the same diet was continued until weaning. Thereafter, both offspring of obese (HFD_Fem & HFD_Mal) and lean mothers (ND_Fem & ND_Mal) were fed a normal diet (left). Weight of dams before mating (right). (B) Weight curve of female (left) and male (right) offspring with weekly weight measurements. (C) Fat mass was measured at 8 (left) and 40 weeks (right) by EchoMRI. (D) Serum level of aspartate aminotransferase (left) and alanine transaminase at 40 weeks (right). (E) Relative composition of the gut microbiome of 40-week-old female offspring at the phylum level was determined by 16S ribosomal RNA gene sequencing, processed with DADA2 pipeline and Silva v138 database (left) and PCA of the proportions of the different bacterial families, based on the 16S data (right). The figure demonstrates clustering according to obesity status of the mother in female offspring at 40 weeks. An ellipse is drawn around the points of each group using the Khachiyan algorithm. (F) The proportion of Bacteroidota and Actinobacteriota species in the offspring of obese and lean mothers as illustrated by boxplots. Data presented as median ± IQR (A, B, D, F) or mean ± SE (C) one dot represents one animal (A, C, D, F), level of significance of $p = 0.05$. Statistical analysis was performed by Wilcoxon–Mann–Whitney test. ND_Fem: Female offspring born to lean mothers n = 6-15, F_HFD: Female offspring born to obese mothers n = 6-10, M_ND male offspring born to lean mothers n = 5-13, M_HFD male offspring born to obese mothers n = 5-14. HFD, high-fat diet; ND, normal diet.

activated by microbe-associated molecular patterns and induce innate immune responses. In the liver, TLR2 and TLR4 are expressed in response to exposure to bacterial products such as bacterial lipopeptides and lipopolysaccharide stemming from the gut.[32] The exposure to maternal obesity during early life led to an overexpression of both TLR2 (ddCt 1.91 *vs.* 0.95, $p = 0.0017$) and TLR4 (ddCt 1.33 *vs.* 1.03, $p = 0.011$) in the liver (Fig. 3F). Likewise, markers of innate immunity such as MHC class II

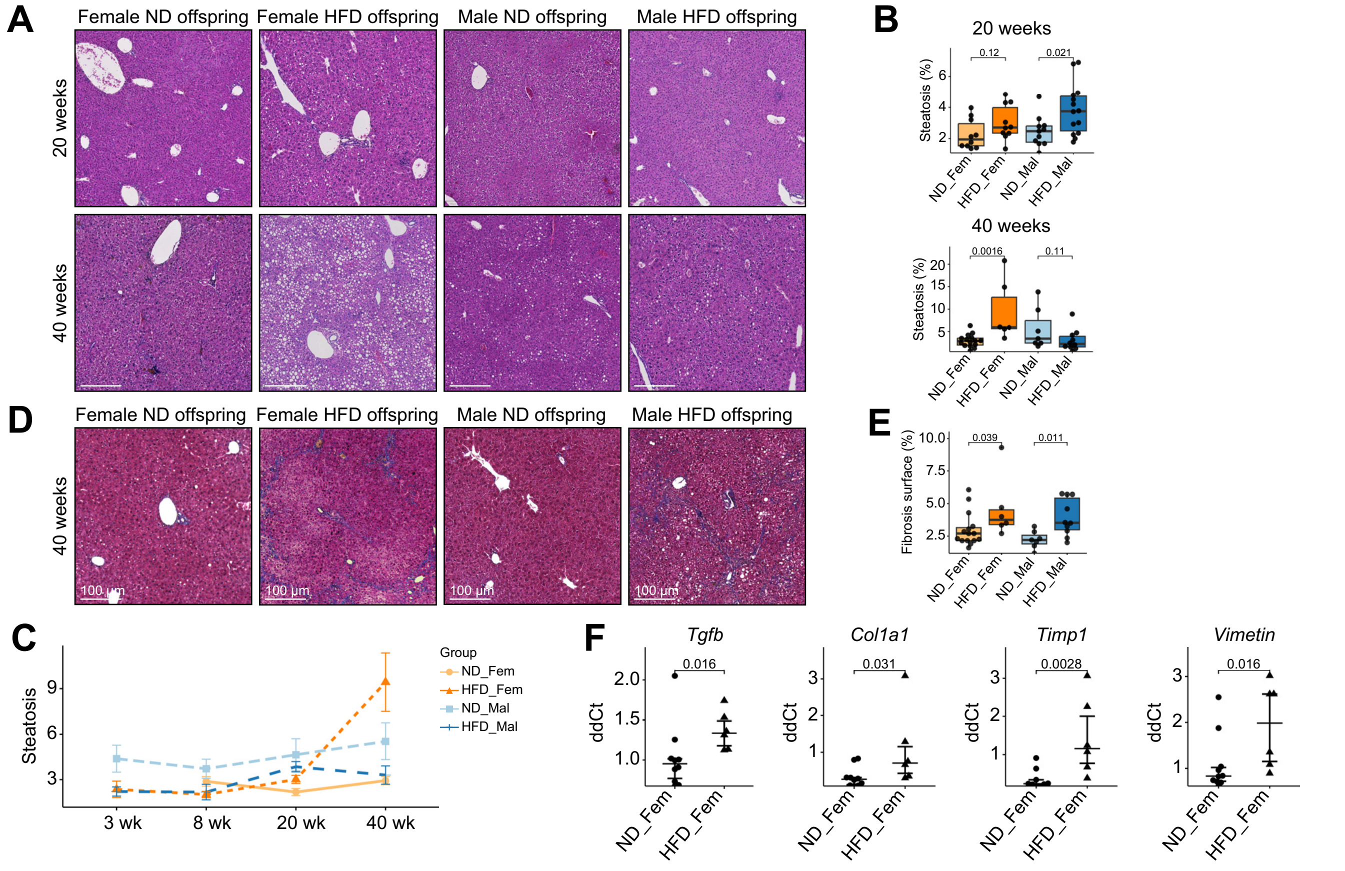


**Fig. 2. Maternal obesity predisposes to the development of steatosis and fibrosis in adult life** (A) Representative H&E-stained histological liver sections from different time points for HFD and ND offspring. (B) Automated quantitative analysis of steatotic tissue was performed. (C) Evolution of steatosis over time. (D) Representative Masson-Trichrome-stained histological liver sections from different time points for HFD and ND offspring. (E) Automated quantitative analysis of fibrotic tissue was performed. (F) Expression of fibrosis markers in liver tissue of female offspring at 40 weeks. Data presented as median ± IQR (B, E, F) or mean ± SE (C) one dot represents one animal (B, E, F), level of significance of $p = 0.05$. Statistical analysis was performed by Wilcoxon–Mann–Whitney test. ND_Fem: Female offspring born to lean mothers n = 6-15, F_HFD: Female offspring born to obese mothers n = 6-10, M_ND male offspring born to lean mothers n = 5-13, M_HFD male offspring born to obese mothers n = 5-14. Scale bar Fig. 2A and 2D: 100 μm. HFD, high-fat diet; ND, normal diet.

molecules, iNOS (inducible nitric oxide synthase) and MCP-1 (monocyte chemoattractant protein-1) were overexpressed in HFD offspring (Fig. 3G). Finally, the chemokine CXCL16, which is expressed by cells of the innate immune system and leads to the recruitment of activated T cells, was expressed at higher levels in the livers of female HFD offspring (Fig. 3G).

In the context of increased liver inflammation, we also performed a detailed analysis of intestinal mucosal inflammation and permeability (Fig. S2). We observed no difference in Lyve-1, a marker for intestinal inflammation and dendritic cell trafficking (Fig. S2A), or alterations in Paneth cell morphology or density, as demonstrated by IHC staining of lysozyme-positive cells for female offspring (Fig. S2B). A HFD induces microbiota-driven disruption of the gut-vascular barrier characterized by an increased accessibility of PV-1 (plasmalemmal vesicle associated protein 1). We did not observe an increased exposure of PV-1 that would indicate a disruption of the gut-vascular barrier in any of the groups tested at 40 weeks (Fig. S2C).

## Maternal obesity increases the risk of developing HCC in a carcinogen-induced liver cancer model

To evaluate whether the increased liver inflammation and steatosis in the HFD offspring translated into a higher risk of liver cancer, we induced hepatic carcinogenesis in the HFD offspring through DEN. The propensity to develop HCC was assessed through micro-CT imaging and serum biomarkers, including alpha-fetoprotein (AFP) at multiple time points (Fig. 4A). DEN is mainly metabolized through cytochrome P450 2E1 in the liver.[33] We observed no difference in the expression of cytochrome P450 2E1 in the livers of the HFD and ND offspring that could have influenced DEN breakdown (Fig. 4B). We observed a trend towards lower liver attenuation on micro-CT imaging at the age of 24 weeks in female HFD offspring (-240 *vs.* -135 HU, $p = 0.054$) indicating a higher liver fat content in female HFD offspring (Fig. 4C), in line with our previous findings. Tumor characteristics such as size and number were determined by manually contouring liver tumors on the axial plane and automatically reconstructing tumor 3D volume (Fig. 4D). Histopathological analysis revealed that most tumors were well-differentiated HCCs, with 10% of tumors in male HFD offspring showing moderate differentiation (Fig. 4E). We observed no differences in terms of microvascular invasion, fatty changes, lymphocyte infiltration, or the presence of pale bodies among the different groups. A higher proportion of male offspring developed tumors compared to female offspring (76.7 *vs.* 58.3%, $p = 0.158$), an observation that has been described

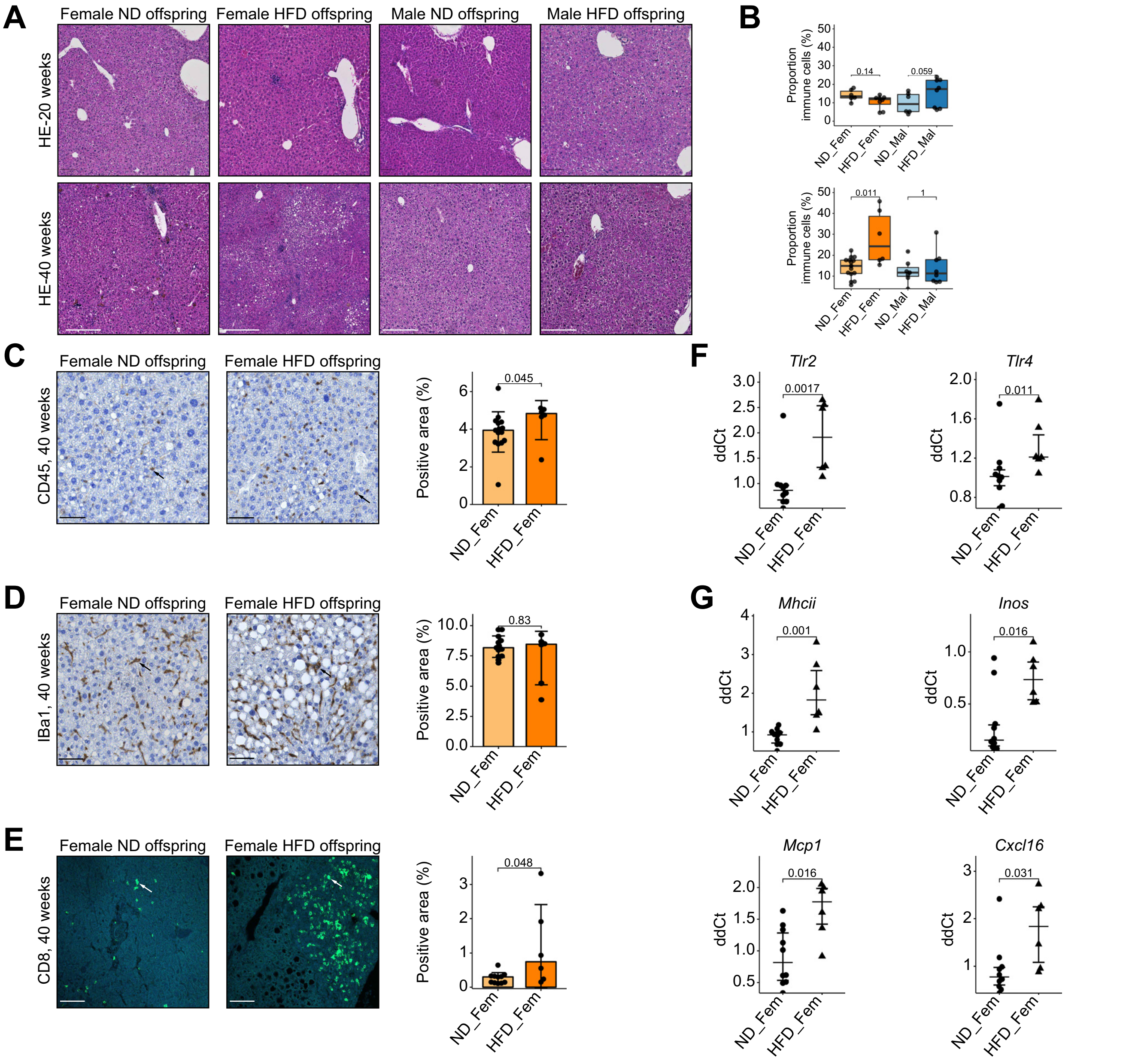


**Fig. 3. Maternal obesity promotes liver inflammation** (A) Representative H&E-stained histological liver sections with immune cell infiltrates. (B) Automated quantitative analysis of tissue surface covered by immune infiltrates. Representative immunofluorescence liver sections of female offspring with corresponding automated quantification (C) CD45, (D) Iba1 and (E) CD8 staining. (F) Expression of TLR family members TLR2 and TLR4 in liver tissue of female offspring at 40 weeks. (G) Expression of inflammatory markers in liver tissue of female offspring at 40 weeks. Data presented as median ± IQR, one dot represents one animal, level of significance of $p = 0.05$. Statistical analysis was performed by Wilcoxon–Mann–Whitney test. Statistical analysis: Wilcoxon signed-rank test (B-G). Median ± IQR and mean ± SE for panel F and G. ND_Fem: Female offspring born to lean mothers n = 6-15, F_HFD: Female offspring born to obese mothers n = 6-10, M_ND male offspring born to lean mothers n = 5-13, M_HFD male offspring born to obese mothers n = 5-14. Scale bar Fig. 2A: 100 µm, 2C-E: 50 µm. HFD, high-fat diet; ND, normal diet.

previously in the DEN model.[34] Among female HFD offspring, a higher proportion developed liver tumors after DEN induction (79.2 *vs.* 37.5%, $p = 0.0084$) with a significantly higher proportion exhibiting ≥3 tumors compared to ND offspring (37.5% *vs.* 4.2%, $p = 0.013$) (Fig. 4F).

Female HFD offspring also had a significantly higher number of tumors (2 *vs.* 0, $p < 0.001$) (Fig. 4G) and a higher mean total tumor volume at 36 weeks than female ND offspring (234 *vs.* 3 $\mu m^3$, $p = 0.0041$) with no significant differences observed for male offspring (332 *vs.* 64 $\mu m^3$, $p = 0.6$) (Fig. 4H). These trends in tumor volume at 36 weeks were also true at 24 and 40 weeks. Furthermore, female HFD offspring exhibited a faster median tumor growth (0.99 *vs.* 0.10 $\mu m^3$/week, $p = 0.006$) (Fig. 4I). AFP is the most widely used serum biomarker in HCC and may reflect tumor burden and aggressiveness.[35] Both male (1,516 *vs.* 258 ng/ml $p = 0.023$) and female HFD offspring (223 *vs.* 0.1 ng/ml, $p < 0.001$) exhibited higher serum levels of AFP at 36 weeks (Fig. 4J).

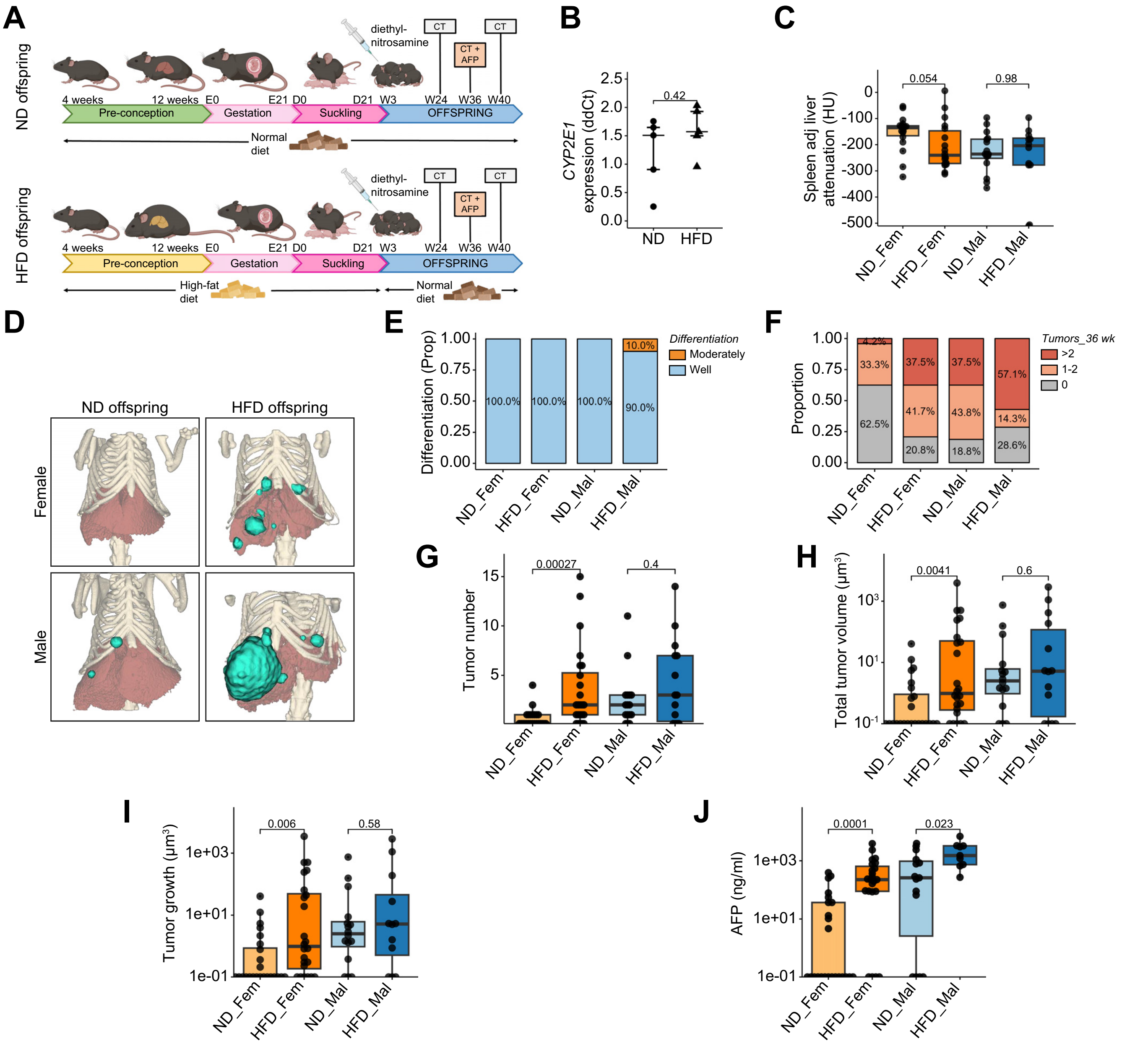

**Fig. 4. Maternal increases the risk of HCC in offspring** (A) Experimental flowchart, dams were fed either a HFD or a ND for 12 weeks before mating, the same diet was continued until weaning. Oncogenesis was induced through injection of DEN, a hepatic carcinogen at 14 days of age. CT scan was performed at weeks 24, 36 and 40. (B) Expression of the main metabolizer of DEN, CYP2E1, in the livers of offspring at weaning. (C) Spleen-adjusted liver attenuation measured on micro-CT images obtained at 24 weeks. (D) Representative 3D reconstructions of liver and tumor volume from micro-CT images obtained at 36 weeks. (E) Tumor differentiation as assessed by an expert liver pathologist. (F) Proportion of animals that developed either no (grey), 1-2 (light red) or >2 tumors (dark red). (G) Tumor number at 36 weeks of age. (H) Total tumor volume at 36 weeks and (I) tumor growth per week. (J) AFP levels determined by ELISA at 36 weeks of age. Data presented as median ± IQR (B, C, G, H, I, J), one dot represents one animal, level of significance of $p$ = 0.05. Statistical analysis was performed by Wilcoxon–Mann–Whitney test. ND: Offspring at weaning born to lean mother n=5, HFD: Offspring at weaning born to obese mothers n=5, ND_Fem: Female offspring born to lean mothers n = 24, F_HFD: Female offspring born to obese mothers n = 24, M_ND male offspring born to lean mothers n = 16, M_HFD male offspring born to obese mothers n = 14. DEN, N-diethylnitrosamine; HCC, hepatocellular carcinoma; HFD, high-fat diet; ND, normal diet.

In a different, transgenic HCC model, where a common oncogene in the pathogenesis of HCC was conditionally overexpressed in the liver (Fig. S3A), we did not observe differences in tumor number, volume or growth in the HFD offspring at the age of 16 weeks (Fig. S3B-E). This indicates that steatotic changes and liver inflammation occurring during adulthood after week 16 play an essential role in the increased risk of HCC in HFD offspring.

### Co-housing of offspring of obese and lean mothers normalizes the gut microbiome

We have shown that the microbiota of offspring born to obese mothers significantly differs from that of offspring born to lean mothers (Fig. 1E) and that the former also face increased risk of developing liver cancer (Fig. 4D). Multiple clinical cohorts have indicated that an altered microbiome is associated with the progression of chronic liver disease.[36,37] Additionally, it is

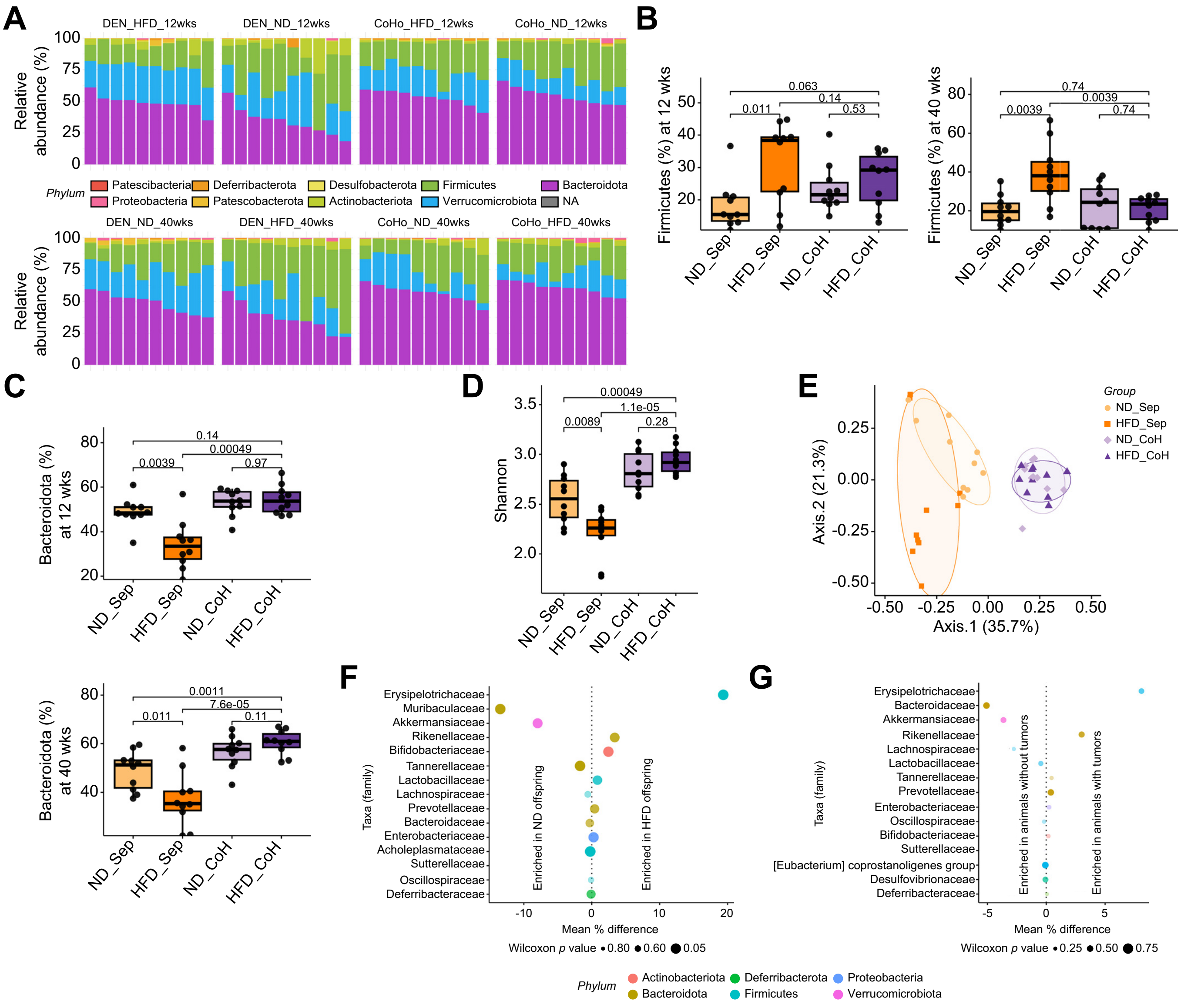

**Fig. 5. Co-housing normalizes the gut microbiome of offspring of obese mothers** Offspring of obese and lean mothers were either housed separately (ND_Sep and HFD_Sep) or co-housed (ND_CoH and HFD_CoH) after weaning. (A) Composition of the gut microbiome of 12-week-old (upper panel) and 40-week-old female (lower panel) offspring was determined at the phylum level by 16S ribosomal RNA gene sequencing. (B) The proportion of Firmicutes and (C) Bacteroidota ASV in offspring of obese and lean mothers is displayed as boxplots. (D) The Shannon diversity index reflects the number of different ASV detected in the stool samples at 40 weeks. (E) PCoA of Bray-Curtis distances of ASV demonstrates clustering according to housing status and diet of the mother in female offspring at 40 weeks. An ellipse is drawn around the points of each group using the Khachiyan algorithm. (F) Comparison of the abundance of microbiota families between separately housed HFD and ND offspring by repeated Wilcoxon test. (G) Comparison of the abundance of microbiota families between offspring that developed tumors or not. The difference in relative abundance is displayed on the x-axis, the size of the dot is representative of the uncorrected *p*-value and the color corresponds to the phylum of the family. Data presented as median ± IQR if not specified otherwise (B, C, D), one dot represents one animal, level of significance of $p = 0.05$. Statistical analysis was performed by Wilcoxon–Mann–Whitney test (B, C, D). n = 10 for all groups. ASV, amplicon sequence variants; HFD, high-fat diet; ND, normal diet; PCoA, principal coordinates analysis.

generally accepted that co-housing of mice normalizes the microbiota between individuals through coprophagy and grooming[38] (Fig. 6A). We, therefore, analyzed the microbiota of separately housed and co-housed HFD and ND offspring. Hepatic carcinogenesis was induced at 14 days of age with DEN. Offspring of all groups received ND after weaning and throughout the experiment. Stool pellets were collected at maturity at 12 weeks and before sacrifice at 40 weeks. We only included female offspring in this co-housing experiment, given that we observed a stronger effect of maternal obesity on the risk for liver cancer in female offspring (Fig. 4D).

The 16S-DNA microbiota profiling generated an average of 50’485 reads per sample (min: 45’526, max: 101’301). Among these, 591 unique ASVs were identified, and were classified at six taxonomic levels using the Silva v138 database.[27] Overall, the gut microbiota was dominated by three phyla: Bacteroidota, Firmicutes and Verrucomicrobiota (Fig. 5A). A higher proportion of Firmicutes has previously been described in some but not all

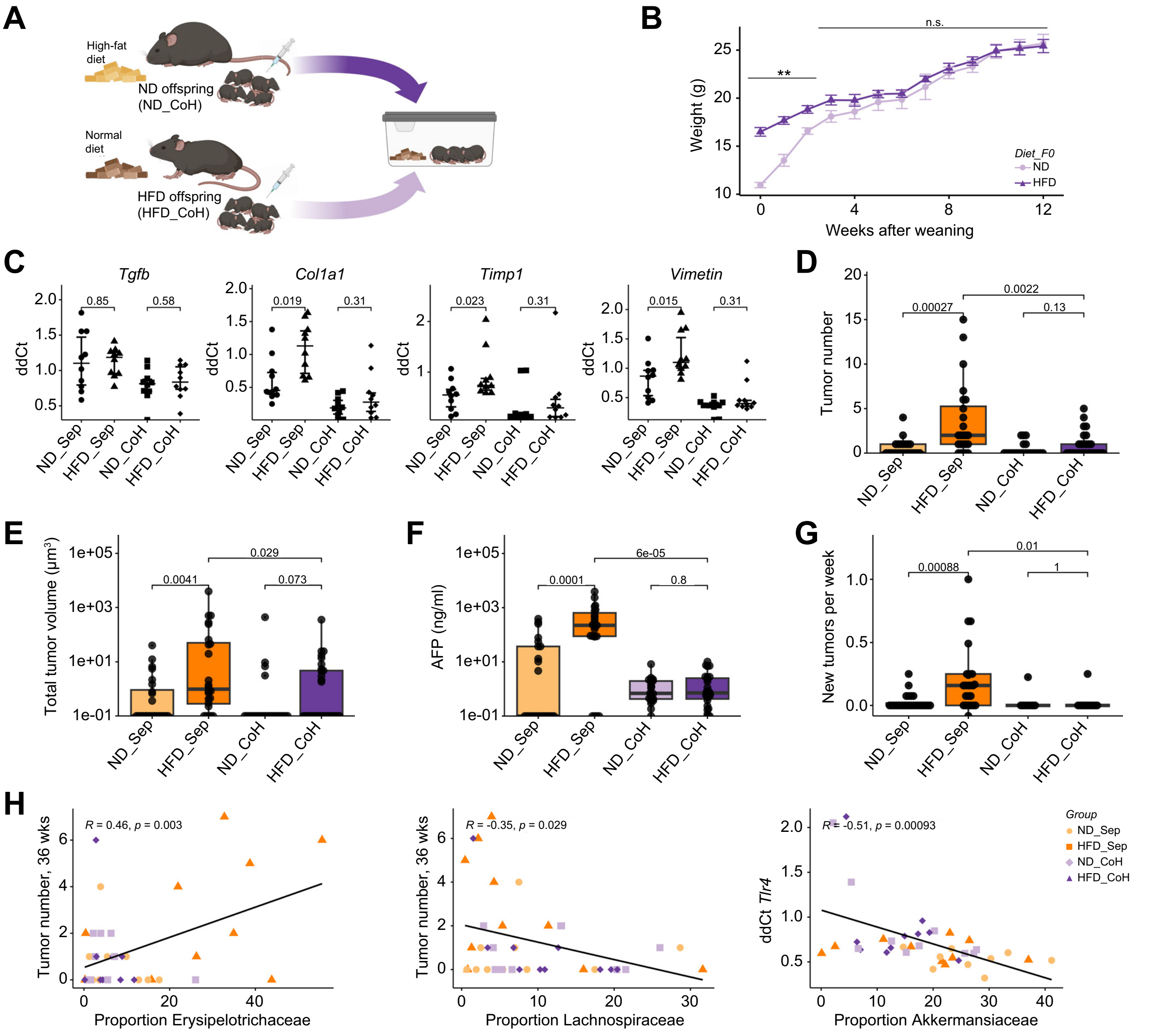


**Fig. 6. Correction of maternal obesity-induced gut dysbiosis by co-housing normalizes HCC risk** (A) Offspring of obese and lean mothers were either housed separately (ND_Sep and HFD_Sep) or co-housed (ND_CoH and HFD_CoH) after weaning. (B) Weight after weaning for the first 12 weeks of life. (C) Expression of pro-fibrotic markers in liver tissue at 40 weeks. Liver tumor mass was assessed by micro-CT at 32 and 36 weeks of age. (D) Tumor number and (E) total tumor volume at 36 weeks of age. (F) Dosing of alpha-fetoprotein in the sera at 36 weeks of age. (G) Number of new tumors normalized by week. (H) Correlation of tumor number or TLR4 expression with the microbiota composition. Data presented as median ± IQR (B, C, D, E, F, G), one dot represents one animal, level of significance of $p$ = 0.05, $p$ >0.05 n.s., $p$ <0.05*, p<0.01**. Statistical analysis was performed by Wilcoxon–Mann–Whitney test (B-G) and pearson's correlation coefficient (H). ND_Sep: Female offspring born to lean mothers housed separately n = 24, HFD_Sep: Female offspring born to obese mothers housed separately n = 24, ND_CoH female offspring born to lean mothers co-housed with offspring of obese mothers n = 24, HFD_CoH female offspring born to obese mothers co-housed with offspring of lean mothers n = 29, correlation plots (H) n = 10 per group. HCC, hepatocellular carcinoma; HFD, high-fat diet; ND, normal diet.

studies of obese individuals and patients with MASLD.[36,39] We found a significantly increased proportion of Firmicutes in HFD offspring compared to ND offspring (38.4 *vs.* 15.5%, $p$ = 0.011). Co-housing of HFD and ND offspring led to a normalization of the proportion of Firmicutes species in the co-housed HFD offspring at 40 weeks, which was comparable to the levels in separately housed ND offspring (23.5 *vs.* 24.4%, $p$ = 0.74) (Fig. 5B). These effects were more pronounced at 40 compared to 12 weeks (Fig. 5B).

ASV from the Bacteroidota phylum were more abundant in co-housed HFD offspring compared to separately housed HFD offspring as early as 12 weeks after birth (53.7 *vs.* 33.5%, $p$ <0.001), which remained constant at 40 weeks (60.9 *vs.* 35.4%, $p$ <0.001) (Fig. 5C). HFD offspring had a significantly less diverse microbiota than ND offspring (2.26 *vs.* 2.56, $p$ = 0.0089) and co-housing of HFD with ND offspring restored the microbial diversity (mean Shannon index ~2.94) (Fig. 5D). We used the ASV-level data to perform a principal coordinate analysis. The latter displays

samples relative to each other based on their differences in terms of presence/absence and relative proportions of all identifiable ASV. Co-housing resulted in a similar microbial composition regardless of maternal diet (Fig. 5E).

To identify the microbes potentially responsible for the increased oncogenesis in HFD offspring, we compared the relative proportions of all represented bacterial families, between the separately housed HFD and ND offspring (Fig. 5F), and offspring that did or did not develop tumors (Fig. 5G). A member of the Firmicutes phylum, the *Erysipelotrichaceae* family, exhibited the largest enrichment in both HFD offspring compared to ND offspring and animals which developed tumors compared to animals without tumors. The *Erysipelotrichaceae* family has previously been shown to increase in abundance under a Western-style diet[40] and a member of the Erysipelotrichaceae family has been identified to be highly immunogenic.[41] On the other hand, *Akkermansiaceae*, a bacterial family previously associated with improved response to immune checkpoint inhibitor and decreased hepatic inflammation was depleted in both HFD offspring and animals that developed tumors.[42,43]

Several other families within the Firmicutes phylum appeared to be driving the differences between the HFD and ND offspring. After false-discovery-rate correction, *Eubacterium coprostanoligenes* remained significantly different in ND and HFD offspring (Fig. 5F). The *Eubacterium* genus was present in all ND offspring, albeit at low abundance (0.05 to 0.25% of all ASV), whereas it was almost totally absent from the HFD offspring (only detectable in 2/10 mice, and lower than 0.05% abundance). *Eubacterium coprostanoligenes* is implicated in cholesterol metabolism by the gut flora and reduces cholesterol to coprostanol.[44] The taxonomy derived from our data and the Silva v138 database did not enable reliable assessment of the genus level.

### Normalization of the gut microbiome normalizes liver cancer risk in a carcinogen-induced DEN model

Next, we analyzed the impact of the correction of the microbial gut flora through co-housing on the tumor load in co-housed offspring in a DEN-induced liver cancer model as described previously (Fig. 6A). Co-housing of female offspring normalized metabolism within 3 weeks after weaning. While the HFD offspring weighed significantly more after weaning (16.5 *vs.* 11.0 g, $p$ <0.001), this difference gradually disappeared over 3 weeks, and co-housed HFD offspring weighed the same as ND offspring by week 5 after weaning (20.8 *vs.* 19.5 g, $p$ = 0.66) (Fig. 6B). Additionally, we observed a normalization of the expression of pro-fibrotic markers in the livers of co-housed HFD offspring compared to separately housed offspring (Fig. 6C). Co-housing did not affect the expression of inflammatory markers (Fig. S4A). Co-housing HFD offspring with ND offspring not only normalized the gut microbiome (Fig. 5D), weight, and pro-fibrotic marker expression but also normalized tumor number. The median number of tumors in separately housed offspring reduced from two to none in co-housed animals ($p$ = 0.0022) (Fig. 6D). The mean tumor volume also decreased in co-housed offspring (234 $\mu m^3$ *vs.* 16 $\mu m^3$, $p$ = 0.029) (Fig. 6E). We confirmed the findings on tumor burden by measurement of AFP, which was significantly decreased in co-housed HFD offspring compared to their separately housed offspring (median AFP 0.7 *vs.* 223 ng/ml, $p$ <0.001) (Fig. 6F). Additionally, co-housed animals exhibited lower levels of carcinogenesis with a significantly lower number of new tumors per week than observed in separately housed animals (0.0 *vs.* 0.16, $p$ = 0.01) (Fig. 6G).

Next, we correlated the composition of the gut microbiota with the expression of inflammatory and pro-fibrotic markers and the risk of developing liver tumors in co-housed and separately housed offspring (Fig. 6H). Members of the *Akkermansiaceae* family were enriched in the microbiome of animals without tumors (Fig. 5H). The *Erysipelotrichaceae* family was the most differentially abundant family between HFD and ND offspring and its abundance is correlated with higher tumor load (Pearson's r 0.46, $p$ = 0.003). On the other hand, a lower abundance of members from the *Lachnospiraceae* family correlated with a higher tumor number (Pearson's $r$ -0.35, $p$ = 0.029). We also found a significant correlation between abundance of *Akkermansiaceae* family and expression of TLR4 in the liver (Pearson's r -0.51, $p$ <0.001).

## Discussion

Our study confirms the negative impact of maternal obesity on the risk of chronic liver disease in offspring during adulthood. Specifically, we found that a Western-style diet in the mother leads to a higher incidence and increased growth of HCC in offspring (Fig. 4G,I). We have shown that maternal obesity alters the microbial composition of the gut flora and that this altered gut microbiota can be corrected through the co-housing of HFD and ND offspring. Remarkably, normalization of the gut microbiome led to a correction of the increased HCC risk among HFD offspring. Our study thus provides mechanistic insight into the transmission of an altered gut microbiota from mother to offspring and its association with increased risk of liver cancer in the offspring of obese mothers. Further validation through clinical and epidemiological studies will help to confirm our findings.

Previous murine studies have shown that maternal obesity is a risk factor for MASLD in offspring.[13,14,45] Hagstrom *et al.* recently confirmed these findings in a large Swedish case-control study.[17] However, these studies suffer from a major shortcoming. The incidence of MASLD increases with age and reaches its peak incidence only in the seventh decade of life.[3] Previous studies have looked at the incidence of steatosis in early adulthood, before the age of 24 weeks in murine studies, or in young adults before the age of 25 years in the case of Hagstrom *et al.* Our findings suggest that steatosis and hepatic inflammation only develop at 40 weeks, with little to no changes before the age of 20 weeks in mice. Additionally, in previous studies, liver injury was heightened by feeding a HFD to offspring of obese mothers. Our results show that even on a control diet, the offspring of obese mothers suffer from a higher risk of chronic liver disease, with steatosis being increased most notably in females. If confirmed in human studies, this would mean that children of obese mothers would require therapy and follow-up beyond dietary interventions to prevent the development of chronic liver disease.

Sun *et al.* recently studied the effect of maternal obesity on the risk of developing HCC using the same liver cancer model employed in our study.[21] Our study validates their findings, revealing that offspring of obese mothers exhibit a higher incidence of liver tumors compared to offspring of non-obese mothers. To explain the underlying mechanism behind this observed increase in oncogenesis, the authors studied gene expression profiles. Their findings highlighted the gradual downregulation of two metabolic genes, *Acsl1* and *Aldh2*, in the offspring of obese mothers. The study linked the downregulation of these genes to an increase of a specific microRNA, miR-27a-3p, which silences the expression of *Acsl1* and *Aldh2*. The

administration of a miR-27a-3p agomir into pregnant mothers significantly increased the tumor load in the offspring. The authors provided compelling evidence on the role of *Acsl1* and *Aldh2* in the disease process. We recently studied the entire genome expression patterns in the livers of offspring born to obese mothers.[16] Using 11 previously published datasets, we did not find these genes to be downregulated.[22] Although regulatory microRNA might play a role in the transmission of liver disease from mother to offspring, it is unlikely to be solely responsible for the observed effect.

Previous studies have also highlighted the role of vertical transmission of the gut microbiome in the development of chronic liver disease in offspring. Soderborg *et al.* demonstrated that germ-free mice colonized with stool microbes of infants of obese mothers developed periportal inflammation and increased intestinal permeability.[24] The microbial composition of the infants of obese mothers was characterized by an increased proportion of Firmicutes and decreased abundance of Bacteroidota. We confirm these clinical findings in our study with similar shifts in the microbial composition (Fig. 5A), consistent with recently published studies.[31,41,44] Interestingly, we found that the altered microbial composition inherited at birth is conserved over the entire life span. Deleterious factors such as increased abundance of Firmicutes or reduced microbial diversity are more pronounced at 40 compared to 12 weeks (Fig. 5B,D). We observed that the offspring of obese mothers have a less diverse gut microbiota, but that diversity can be restored through co-housing with the offspring of lean mothers. This aligns with clinical studies that have shown that increased microbial diversity is associated with a lower risk of decompensation in patients with cirrhosis[46] or advanced fibrosis/cirrhosis in patients with MASLD.[36,47,48]

Clinical studies suggest that gut-mediated hepatic inflammation may contribute to carcinogenesis in patients with HCC.[48,49] In the livers of offspring of obese mothers, we observed an increased number of inflammatory infiltrates that were rich in macrophages and an overexpression of inflammatory markers (Fig. 3). Additionally, immunogenic bacterial strains are enriched in the microbiota of HFD offspring, while bacterial strains associated with decreased hepatic inflammation are depleted (Fig. 6F). Tumor load and abundance of Erysipelotrichaceae had a positive correlation, whereas the abundance of Lachnospiracea exhibited a negative correlation. Previous animal studies have shown that endotoxin accumulation in the portal circulation can promote HCC growth through TLR4 activation.[50,51] We observed a hepatic overexpression of TLR4 in female offspring of obese mothers. We found Akkermansiaceae, a bacterial family previously associated with improved response to immune checkpoint inhibitors, and decreased hepatic inflammation, to be depleted in both HFD offspring and animals that developed tumors.[42,43] The lower proportion of Akkermansiaceae in fecal samples was also linked with increased levels of TLR4 expression. Increased inflammatory signaling induced by an altered gut microbiome profile likely contributed to increased HCC growth in the offspring of obese mothers.

In a mechanistic approach we studied how the altered gut microbiota may influence intestinal inflammation and the gut-vascular barrier and therefore favor the progression of liver disease. We did not observe increased intestinal density of lymphatic vessels or Paneth cells in offspring of obese mothers, nor did we find alterations in the gut-vascular barrier (Fig. S2). Future studies should incorporate additional mechanistic explorations, such as *in vivo* assessments of intestinal permeability, presence of microbial metabolites and microbial-associated molecular patterns in the portal blood, and concentrations of short-chain fatty acids in stool samples, to improve our understanding of how dysbiosis contributes to the progression of liver disease.

Our study has several limitations. To discern the importance of the post-natal *vs.* pre-natal environment on our findings, conducting cross-fostering experiments, where offspring of HFD-fed mothers are transferred to and reared by ND-fed mothers after birth,[13] would have been valuable. Unfortunately, our attempts to reproduce our results in the LAP-Myc carcinogenesis model were unsuccessful (Fig. S3). We posit that the LAP-Myc model induces a tumor growth that is both too early and too aggressive for the specific context of our study, hindering our ability to capture the deleterious impact of the transmission of an altered gut microbiome. Especially, since we did not observe any significant liver injury before 40 weeks of age in HFD offspring (Figs 2 and 3). Furthermore, we observed significant sex disparity in the susceptibility of HFD-fed mice to HCC and liver injury. We speculate that hormonal differences and varying susceptibilities of male and female mice to DEN-induced carcinogenesis play a role.[52] However, only future experiments with detailed analysis of microbiome differences between male and female offspring will provide conclusive answers.

In summary, we describe an important link between maternal obesity, alterations of the gut microbiome, and increased risk of HCC. Given the high prevalence of obesity in mothers and the temporal gap between exposure and tumor development, our observations carry important public health implications for future generations. The implementation of microbial screening and restoration of microbial diversity holds promise in helping to identify and treat individuals at risk. Future studies are imperative to characterize how microbiota influences hepatic carcinogenesis.

## Abbreviations

AFP, alpha-fetoprotein; ASV, amplicon sequence variants; DEN, N-diethylnitrosamine; HCC, hepatocellular carcinoma; HFD, high-fat diet; HFD offspring, offspring of HFD-fed mothers; MASLD, metabolic dysfunction-associated steatotic liver disease; ND, normal diet; ND offspring, offspring of ND-fed mothers; PCA, principal component analysis; PCoA, principal coordinates analysis; TLR, Toll-like receptor.

## Financial support

The Swiss National Science Foundation (grant number 182471), The Fondation Francis & Marie-France Minkoff, Prof. Dr. Max Cloëtta Foundation, and the Leenaards Foundation (grant number 5489) funded this research.

## Conflict of interest

The authors declare that they have no conflict of interest.

Please refer to the accompanying ICMJE disclosure forms for further details.

## Authors' contributions

Conceptualization, B.M., V.D., A.P., G.O., S.L., and C.T.; methodology, B.M., V.D., B.G., R.M., A.G., L.R–B., F.S., Q.G. and S.L.; investigation; data curation,

B.M., V.D., B.G., S.EH., J.R.R., R.M., A.G., L.R–B, F.S., Q.G., S.L.; writing—original draft preparation, B.M., B.G. and S.L.; writing—review and editing, V.D., S.EH., A.P., G.O., S.L. and C.T.; supervision, S.L. and C.T.; project administration, S.L. and C.T.; funding acquisition, B.M., S.L. and C.T. All authors have read and agreed to the published version of the manuscript.

## Data availability statement

All data utilized in this manuscript is accessible and has been deposited in the publicly available archive with the following DOI: https://doi.org/10.26037/yareta:ef2h6hlu5zc3zhw6fiuywsj54m. Researchers and readers are encouraged to refer to this repository for access to the complete dataset supporting our findings.


## Acknowledgments

We thank the personnel of Microbiome Insights for the 16S rRNA gene sequencing. The Genomic Core Facility of the Faculty of Medicine (University of Geneva), in particular Christelle Barraclough for their help in qRT-PCR. The authors also thank the Histology Core Facility of the Faculty of Medicine (University of Geneva) and Nicolas Liaudet of the Bioimaging core facility of the Faculty of Medicine (University of Geneva). Additionally, we express gratitude to Ilaria Di Meglio for her valuable assistance in English language editing of the manuscript.


## Supplementary data

Supplementary data to this article can be found online at https://doi.org/10.1016/j.jhepr.2024.101056.

## References


[1] Ng M, Fleming T, Robinson M, et al. Global, regional, and national prevalence of overweight and obesity in children and adults during 1980–2013: a systematic analysis for the Global Burden of Disease Study 2013. The Lancet 2014;384:766–781. https://doi.org/10.1016/S0140-6736(14)60460-8.

[2] Hales CM. Prevalence of obesity and severe obesity among adults: United States, 2017–2018. 2020. p. 8.

[3] Quek J, Chan KE, Wong ZY, et al. Global prevalence of non-alcoholic fatty liver disease and non-alcoholic steatohepatitis in the overweight and obese population: a systematic review and meta-analysis. The Lancet Gastroenterol Hepatol 2022. https://doi.org/10.1016/S2468-1253(22)00317-X. 0.

[4] Rinella ME, Lazarus JV, Ratziu V, et al. A multisociety Delphi consensus statement on new fatty liver disease nomenclature. Hepatology n.d. https://doi.org/10.1097/HEP.0000000000000520.

[5] Singh S, Allen AM, Wang Z, et al. Fibrosis progression in nonalcoholic fatty liver *vs.* nonalcoholic steatohepatitis: a systematic review and meta-analysis of paired-biopsy studies. Clin Gastroenterol Hepatol 2015;13:643–654. https://doi.org/10.1016/j.cgh.2014.04.014. e1-9;quiz e39-40.

[6] Simon TG, Roelstraete B, Sharma R, et al. Cancer risk in patients with biopsy-confirmed nonalcoholic fatty liver disease: a population-based cohort study. Hepatology 2021;74:2410–2423. https://doi.org/10.1002/hep.31845.

[7] Orci LA, Sanduzzi-Zamparelli M, Caballol B, et al. Incidence of hepatocellular carcinoma in patients with nonalcoholic fatty liver disease: a systematic review, meta-analysis, and meta-regression. Clin Gastroenterol Hepatol 2022;20:283–292.e10. https://doi.org/10.1016/j.cgh.2021.05.002.

[8] Sung H, Ferlay J, Siegel RL, et al. Global cancer statistics 2020: GLOBOCAN estimates of incidence and mortality worldwide for 36 cancers in 185 countries. CA: A Cancer J Clinicians 2021;71:209–249. https://doi.org/10.3322/caac.21660.

[9] Heslehurst N, Vieira R, Akhter Z, et al. The association between maternal body mass index and child obesity: a systematic review and meta-analysis. PLOS Med 2019;16:e1002817. https://doi.org/10.1371/journal.pmed.1002817.

[10] Massion S, Wickham S, Pearce A, et al. Exploring the impact of early life factors on inequalities in risk of overweight in UK children: findings from the UK Millennium Cohort Study. Arch Dis Child 2016;101:724–730. https://doi.org/10.1136/archdischild-2015-309465.

[11] Adane AA, Mishra GD, Tooth LR. Maternal pre-pregnancy obesity and childhood physical and cognitive development of children: a systematic review. Int J Obes (Lond) 2016;40:1608–1618. https://doi.org/10.1038/ijo.2016.140.

[12] Radford-Smith DE, Anthony DC. Mechanisms of maternal diet-induced obesity affecting the offspring brain and development of affective disorders. Metabolites 2023;13:455. https://doi.org/10.3390/metabo13030455.

[13] Oben JA, Mouralidarane A, Samuelsson AM, et al. Maternal obesity during pregnancy and lactation programs the development of offspring non-alcoholic fatty liver disease in mice. J Hepatol 2010;52:913–920. https://doi.org/10.1016/j.jhep.2009.12.042.

[14] Mouralidarane A, Soeda J, Visconti-Pugmire C, et al. Maternal obesity programs offspring nonalcoholic fatty liver disease by innate immune dysfunction in mice. Hepatology 2013;58:128–138. https://doi.org/10.1002/hep.26248.

[15] Bruce KD, Cagampang FR, Argenton M, et al. Maternal high-fat feeding primes steatohepatitis in adult mice offspring, involving mitochondrial dysfunction and altered lipogenesis gene expression. Hepatology 2009;50:1796–1808. https://doi.org/10.1002/hep.23205.

[16] Moeckli B, Delaune V, Prados J, et al. Impact of maternal obesity on liver disease in the offspring: a comprehensive transcriptomic analysis and confirmation of results in a murine model. Biomedicines 2022;10:294. https://doi.org/10.3390/biomedicines10020294.

[17] Hagstrom H, Simon TG, Roelstraete B, et al. Maternal obesity increases the risk and severity of NAFLD in offspring. J Hepatol 2021;75:1042–1048. https://doi.org/10.1016/j.jhep.2021.06.045.

[18] Murphy CC, Cirillo PM, Krigbaum NY, et al. Maternal obesity, pregnancy weight gain, and birth weight and risk of colorectal cancer. Gut 2021. https://doi.org/10.1136/gutjnl-2021-325001.

[19] Stacy SL, Buchanich JM, Ma ZQ, et al. Maternal obesity, birth size, and risk of childhood cancer development. Am J Epidemiol 2019;188:1503–1511. https://doi.org/10.1093/aje/kwz118.

[20] Kessous R, Wainstock T, Sheiner E. Pre-pregnancy obesity and childhood malignancies: a population-based cohort study. Pediatr Blood Cancer 2020;67:e28269. https://doi.org/10.1002/pbc.28269.

[21] Sun Y, Wang Q, Zhang Y, et al. Multigenerational maternal obesity increases the incidence of HCC in offspring via miR-27a-3p. J Hepatol 2020;73:603–615. https://doi.org/10.1016/j.jhep.2020.03.050.

[22] Moeckli B, Lacotte S, Toso C. The role of Acsl1 and Aldh2 in the increased risk for liver cancer in offspring of obese mothers. Front Med 2022;9.

[23] Ferretti P, Pasolli E, Tett A, et al. Mother-to-Infant microbial transmission from different body sites shapes the developing infant gut microbiome. Cell Host Microbe 2018;24:133–145.e5. https://doi.org/10.1016/j.chom.2018.06.005.

[24] Soderborg TK, Clark SE, Mulligan CE, et al. The gut microbiota in infants of obese mothers increases inflammation and susceptibility to NAFLD. Nat Commun 2018;9:4462. https://doi.org/10.1038/s41467-018-06929-0.

[25] Callahan BJ, McMurdie PJ, Rosen MJ, et al. DADA2: high-resolution sample inference from Illumina amplicon data. Nat Methods 2016;13:581–583. https://doi.org/10.1038/nmeth.3869.

[26] R Core Team. R: a language and environment for statistical computing. 2021.

[27] Quast C, Pruesse E, Yilmaz P, et al. The SILVA ribosomal RNA gene database project: improved data processing and web-based tools. Nucleic Acids Res 2013;41:D590–D596. https://doi.org/10.1093/nar/gks1219.

[28] McMurdie PJ, Holmes S. Phyloseq: an R package for reproducible interactive analysis and graphics of microbiome census data. PLOS ONE 2013;8:e61217. https://doi.org/10.1371/journal.pone.0061217.

[29] Hadley W. ggplot2: elegant graphics for data analysis. New York: Springer-Verlag; 2016.

[30] Gloor GB, Macklaim JM, Pawlowsky-Glahn V, et al. Microbiome datasets are compositional: and this is not optional. Front Microbiol 2017;8.

[31] Turnbaugh PJ, Ley RE, Mahowald MA, et al. An obesity-associated gut microbiome with increased capacity for energy harvest. Nature 2006;444:1027–1031. https://doi.org/10.1038/nature05414.

[32] Zähringer U, Lindner B, Inamura S, et al. TLR2 - promiscuous or specific? A critical re-evaluation of a receptor expressing apparent broad specificity. Immunobiology 2008;213:205–224. https://doi.org/10.1016/j.imbio.2008.02.005.

[33] Ye Q, Lian F, Chavez PRG, et al. Cytochrome P450 2E1 inhibition prevents hepatic carcinogenesis induced by diethylnitrosamine in alcohol-fed rats. Hepatobiliary Surg Nutr 2012;1:58. https://doi.org/10.3978/j.issn.2304-3881.2012.11.05. 18.

[34] Li Z, Tuteja G, Schug J, et al. Foxa1 and Foxa2 are essential for sexual dimorphism in liver cancer. Cell 2012;148:72–83. https://doi.org/10.1016/j.cell.2011.11.026.

[35] Toso C, Meeberg G, Hernandez-Alejandro R, et al. Total tumor volume and alpha-fetoprotein for selection of transplant candidates with

hepatocellular carcinoma: a prospective validation. Hepatology 2015;62:158–165. https://doi.org/10.1002/hep.27787.
[36] Caussy C, Tripathi A, Humphrey G, et al. A gut microbiome signature for cirrhosis due to nonalcoholic fatty liver disease. Nat Commun 2019;10:1406. https://doi.org/10.1038/s41467-019-09455-9.
[37] Boursier J, Mueller O, Barret M, et al. The severity of nonalcoholic fatty liver disease is associated with gut dysbiosis and shift in the metabolic function of the gut microbiota. Hepatology 2016;63:764–775. https://doi.org/10.1002/hep.28356.
[38] Robertson SJ, Lemire P, Maughan H, et al. Comparison of Co-housing and littermate methods for microbiota standardization in mouse models. Cell Rep 2019;27:1910–1919.e2. https://doi.org/10.1016/j.celrep.2019.04.023.
[39] Ley RE, Turnbaugh PJ, Klein S, et al. Microbial ecology: human gut microbes associated with obesity. Nature 2006;444:1022–1023. https://doi.org/10.1038/4441022a.
[40] Fleissner CK, Huebel N, El-Bary MMA, et al. Absence of intestinal microbiota does not protect mice from diet-induced obesity. Br J Nutr 2010;104:919–929. https://doi.org/10.1017/S0007114510001303.
[41] Palm NW, de Zoete MR, Cullen TW, et al. Immunoglobulin A coating identifies colitogenic bacteria in inflammatory bowel disease. Cell 2014;158:1000–1010. https://doi.org/10.1016/j.cell.2014.08.006.
[42] Grenda A, Iwan E, Chmielewska I, et al. Presence of Akkermansiaceae in gut microbiome and immunotherapy effectiveness in patients with advanced non-small cell lung cancer. AMB Express 2022;12:86. https://doi.org/10.1186/s13568-022-01428-4.
[43] Schneeberger M, Everard A, Gómez-Valadés AG, et al. Akkermansia muciniphila inversely correlates with the onset of inflammation, altered adipose tissue metabolism and metabolic disorders during obesity in mice. Sci Rep 2015;5:16643. https://doi.org/10.1038/srep16643.
[44] Kriaa A, Bourgin M, Mkaouar H, et al. Microbial reduction of cholesterol to coprostanol: an old concept and new insights. Catalysts 2019;9:167. https://doi.org/10.3390/catal9020167.
[45] Wankhade UD, Zhong Y, Kang P, et al. Maternal high-fat diet programs offspring liver steatosis in a sexually dimorphic manner in association with changes in gut microbial ecology in mice. Sci Rep 2018;8:16502. https://doi.org/10.1038/s41598-018-34453-0.
[46] Bajaj JS, Idilman R, Mabudian L, et al. Diet affects gut microbiota and modulates hospitalization risk differentially in an international cirrhosis cohort. Hepatology 2018;68:234–247. https://doi.org/10.1002/hep.29791.
[47] Qin N, Yang F, Li A, et al. Alterations of the human gut microbiome in liver cirrhosis. Nature 2014;513:59–64. https://doi.org/10.1038/nature13568.
[48] Ponziani FR, Bhoori S, Castelli C, et al. Hepatocellular carcinoma is associated with gut microbiota profile and inflammation in nonalcoholic fatty liver disease. Hepatology 2019;69:107–120. https://doi.org/10.1002/hep.30036.
[49] Ren Z, Li A, Jiang J, et al. Gut microbiome analysis as a tool towards targeted non-invasive biomarkers for early hepatocellular carcinoma. Gut 2019;68:1014–1023. https://doi.org/10.1136/gutjnl-2017-315084.
[50] Dapito DH, Mencin A, Gwak G-Y, et al. Promotion of hepatocellular carcinoma by the intestinal microbiota and TLR4. Cancer Cell 2012;21:504–516. https://doi.org/10.1016/j.ccr.2012.02.007.
[51] Yu L-X, Yan H-X, Liu Q, et al. Endotoxin accumulation prevents carcinogen-induced apoptosis and promotes liver tumorigenesis in rodents. Hepatology 2010;52:1322–1333. https://doi.org/10.1002/hep.23845.
[52] Naugler WE, Sakurai T, Kim S, et al. Gender disparity in liver cancer due to sex differences in MyD88-dependent IL-6 production. Science 2007;317:121–124. https://doi.org/10.1126/science.1140485.

Journal of Hepatology, Volume *6*

## Supplemental information

# Maternal obesity increases the risk of hepatocellular carcinoma through the transmission of an altered gut microbiome

**Beat Moeckli, Vaihere Delaune, Benoît Gilbert, Andrea Peloso, Graziano Oldani, Sofia El Hajji, Florence Slits, Joana Rodrigues Ribeiro, Ruben Mercier, Adrien Gleyzolle, Laura Rubbia-Brandt, Quentin Gex, Stephanie Lacotte, and Christian Toso**

# Maternal obesity increases the risk of hepatocellular carcinoma through the transmission of an altered gut microbiome

Beat Moeckli, Vaihere Delaune, Benoît Gilbert, Andrea Peloso, Graziano Oldani, Sofia El Hajji, Florence Slits, Joana Rodrigues Ribeiro, Ruben Mercier, Adrien Gleyzolle, Laura Rubbia-Brandt, Quentin Gex, Stephanie Lacotte, Christian Toso

Table of contents

Supplementary materials and methods ........................................................ 2

Fig. S1 ........................................................................................................ 6

Fig. S2 ........................................................................................................ 8

Fig. S3 ........................................................................................................ 9

Fig. S4 ........................................................................................................ 11

Supplementary references........................................................................... 12

# Supplementary materials and methods

## Transgenic HCC mouse model

LAP-tTA (LAP, B6.NMRI-Tg(Cebpb-tTA)5Bjd/Cnbc, EM:04498) x TRE MYC (B6.Cg-Tg(tetO-MYC,-OVAL)#Gtgm/Ieg, EM:04319) (LAP-Myc) mice were used as HCC transgenic mouse model. Single transgenic LAP-tTA mothers received a HFD for twelve weeks before mating with lean TRE MYC males. Myc expression in the liver was activated by removing the doxycycline enriched diet (Safe Diet, 0.625g/Kg Doxycycline Hyclate) [1,2]. Offspring were assigned to one of the following groups: Stop of doxycycline at 5 weeks with feeding of normal diet (ND) (Dox5w_F1ND), stop of doxycycline at 5 weeks with feeding of choline-deficient methionine controlled high-fat diet (MCD) (Dox5w_F1MCD), or stop of doxycycline at 3 weeks of age with feeding of a ND (Dox3w_F1ND). All animals were sacrificed at 16 weeks.

## Physiological and metabolic phenotyping

Body composition (fat and fat-free mass) was determined in vivo using quantitative magnetic resonance (EchoMRI™ 3-in-1 v2.1; Echo Medical Systems, Houston, TX) at different time points. Glycemia was determined using an Accu-Check glucometer (Roche Diabetes Care, Rotkreuz, Switzerland). For fasting glycemia, mice were fasted for 6 hours.

## Plasma Analysis

Serum alanine aminotransferase (ALT) and aspartate aminotransferase (AST) levels were analyzed using the cobas c111 clinical chemistry analyzer (Roche Diagnostic, Rotkreuz, Switzerland).

## Liver histopathological analysis and immuno-labelling

Liver samples were processed routinely in 10% neutral buffered formalin for 24 h, and then embedded in paraffin. The fixed liver tissues sliced into 5-μm thickness and

stained with haematoxylin and eosin (H&E) and Masson Trichrome (MT) to evaluate liver histology, tumor histology and grading of liver injury. Leukocytes, Macrophages and CD8 T cells were labelled with an anti-CD45 (clone D3F8Q), an anti-Iba1 (clone EPR16588) or an anti-CD8 (clone D4W2Z) followed by an Alexa488-goat anti-rabbit IgG (A32731, Invitrogen) or the SignalStain® Boost IHC Detection Reagent (HRP anti-rabbit, Cell Signaling Technology).

Liver cells apoptosis and proliferation were assessed with anti-cleaved caspase-3 (clone 5A1E) and anti-Ki-67 (clone D3B5) labelling, followed by the SignalStain® Boost IHC Detection Reagent (HRP anti-rabbit, Cell Signaling Technology). Intestinal inflammation and integrity were assessed with anti-Lyzozyme (clone EPR2994) [3,4], anti-Lyve-1 (goat polyclonal AF2125, R&D Systems) [5] or anti-PLVAP (clone E4U6V) labelling [6], followed by the SignalStain® Boost IHC Detection Reagent (HRP anti-rabbit) or by biotin rabbit anti-goat (E0466, Agilent Dako) and HRP-Streptavidin (P0397, Agilent Dako). Labelling was revealed following incubation with 3,3'-diaminobenzidine tetrahydrochloride (DAB, Pierce) and counterstained with haematoxylin.

## Assessment and Quantification of steatosis, fibrosis and immuno-labelling

Steatosis, fibrosis and immune-labelling quantifications were performed with the QuPath software package (version 0.3.2 and 0.4.3, QuPath, Edinburgh, UK) [7]. Sagittal sections of the median and the left liver lobe (one each, per animal) and axial sections of intestines (four per animal) were included in the analysis. Whole slide images were scanned with the Zeiss Axioscan-Z1 Microscope Slide Scanner (Carl Zeiss AG, Jena, Germany). Parts of the slide containing tissue were detected in QuPath using the *SimpleTissueDetection* function. The thereby created annotations were automatically assessed with the *createAnnotationFromPixelClassifier* function using a trained pixel classifier to only include liver tissue without large vessels or preparation artefacts.

For steatosis, lipid vacuoles were automatically detected and validated visually in H&E stained slides. The relative area of steatosis was calculated as the area of vacuoles normalized by the total area of the whole slide.

Fibrosis, stained in blue in MT slides was automatically quantified using a manually trained pixel classifier, which discriminates fibrosis based on pixel coloration. By applying this threshold, we determined the percentage of fibrosis on the total surface of each section.

Anti-CD45, anti-Iba-1, anti-CD8, anti-Ki67, anti-cleaved caspase- 3, anti-PLVAP were automatically quantified using an object classifier (Ki67, cleaved caspase-3) or using a pixel classifier (CD45, Iba-1, CD8 and PLVAP) manually trained on 3,3′-Diaminobenzidine (DAB) staining. Hepatocytes and immune cells were discriminated using an object classifier manually trained on nuclei size (hematoxylin staining).

## Assessment of intestinal histopathology

The count of Paneth cells was visually conducted on 50 villi per section. Only cells with a clearly visible nucleus and positive staining (lysozyme) throughout the cytoplasm were considered. By dividing the number of counted Paneth cells by the number of vili, the Paneth cell/villus ratio was obtained. This was performed on three sections per specimen. The count of lymphatic vessels (Lyve-1 labelling) was also visually conducted around the entire circumference of the sections. Only lymphatic vessels with a visible lumen and present in the submucosa were counted. By dividing the number of lymphatic vessels by the total number of villi, the lymphatic vessel/villus ratio was obtained. This was performed on three sections per specimen.

## Hepatic gene expression

After total RNA extraction of liver tissue (ReliaPrepTM RNA Tissue, Promega, Madison WI, USA), cDNA was synthesised by extending a mix of random primers with the High Capacity cDNA Reverse Transcription Kit in the presence of RNase inhibitor (Applied Biosystems). The relative quantity of each transcript was normalized to the expression of *Eef1*, *Hprt*, and *Gapdh*. SYBRGreen reagent was used for Real-time PCR on the ABI Prism 7000 sequence detection system (Applied

Biosystems, Waltham MA, United States) according to the manufacturer's instructions. Primer sequences are provided in the Supplementary CTAT Table.

## Reconstruction of 3D tumor volume

3D reconstructions were performed with liver and bone segmentation via the Imalytics software (Imalytics Preclinical 3.0 (Gremse-IT GmbH, Aachen, Germany). 3D rendering was performed after tumor morphological identification by manually circling ROI in 1mm thick, three axial slice views.

# Fig. S1

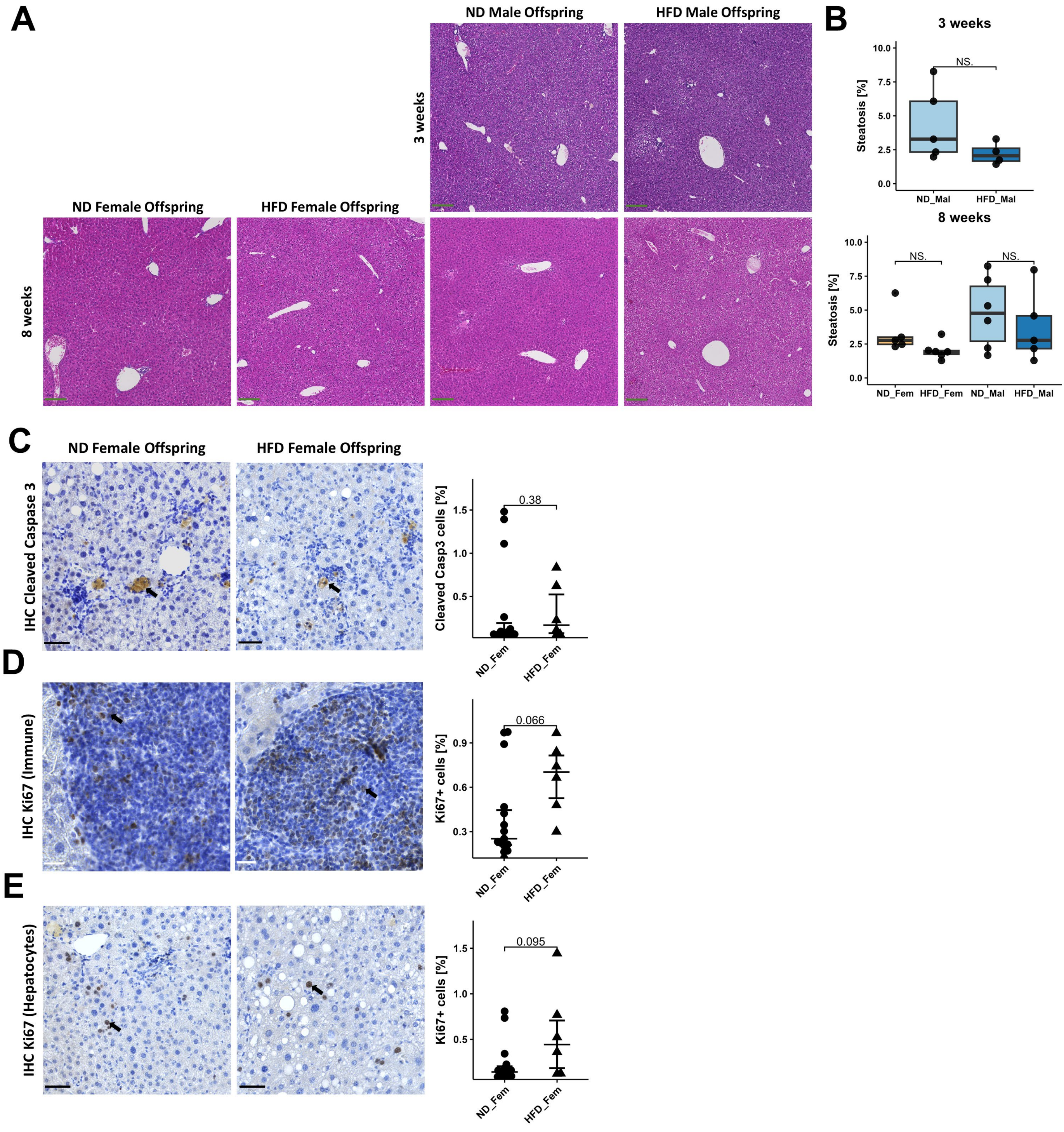
A
ND Male Offspring
HFD Male Offspring
3 weeks
ND Female Offspring
HFD Female Offspring
8 weeks
B
3 weeks
NS.
Steatosis [%]
ND_Mal
HFD_Mal
8 weeks
NS.
NS.
Steatosis [%]
ND_Fem
HFD_Fem
ND_Mal
HFD_Mal
C
ND Female Offspring
HFD Female Offspring
IHC Cleaved Caspase 3
0.38
Cleaved Casp3 cells [%]
ND_Fem
HFD_Fem
D
IHC Ki67 (Immune)
0.066
Ki67+ cells [%]
ND_Fem
HFD_Fem
E
IHC Ki67 (Hepatocytes)
0.095
Ki67+ cells [%]
ND_Fem
HFD_Fem

**Fig. S1. Maternal obesity does not affect steatosis before adulthood and increases compensatory proliferation** Female and male offspring of obese (HFD_Fem & HFD_Mal) and lean mothers (ND_Fem & ND_Mal) were fed a normal diet. (**A**) Histological hematoxylin-eosin stained sections from male offspring at 3 weeks of age, as well as from female and male offspring at 8 weeks of age from ND and HFD mothers. (**B**) Automated quantification of steatosis, expressed as % of liver surface covered by steatotic vesicles. (**C**) Histological sections with immunohistochemistry staining of cleaved caspase 3, (**D**) Ki67 in immune cells, and (**E**) Ki67 in hepatocytes between the two groups with corresponding quantification of stained area. Data presented as median ± IQR, one dot represents one animal, level of significance of p=0.05. Statistical analysis was performed by Wilcoxon–Mann–Whitney test (B, C, D, E). ND_Fem: Female offspring born to lean mothers n = 6-15, F_HFD: Female offspring born to obese mothers n = 6-10, M_ND male offspring born to lean mothers n = 5-7, M_HFD male offspring born to obese mothers n = 5-8. Scale bar Panel A: 100µm, Panels C-E: 50µm.

# Fig. S2

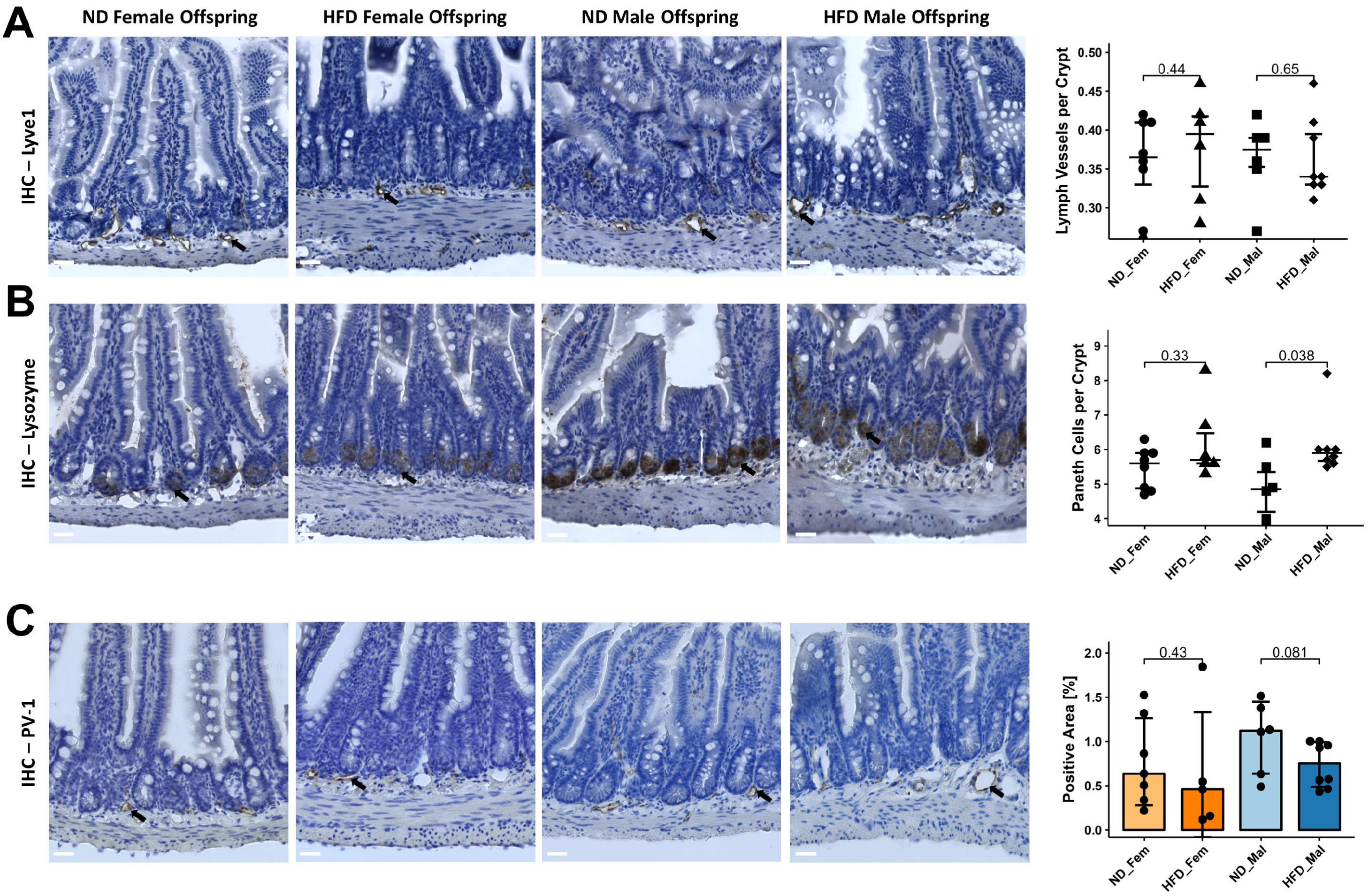


**Fig. S2. Maternal obesity does not affect intestinal inflammation and the gut vascular barrier** Female and male offspring of obese (HFD_Fem & HFD_Mal) and lean mothers (ND_Fem & ND_Mal) were fed a normal diet. (**A**) Axial histological sections of intestines with Immunohistochemistry staining for lymphatic vessels with anti-LYVE-1. Manual quantification of lymph vessels per crypt (right). (**B**) Axial histological sections of intestines with Immunohistochemistry staining for Paneth cells with anti-lysozyme. Manual quantification of Paneth cells per crypt (right). (**C**) Axial histological sections of intestines immunohistochemistry staining for gut vascular barrier with anti-PV-1. Automated quantification of stained area (right). Data presented as median ± IQR, one dot represents one animal, level of significance of p=0.05. Statistical analysis was performed by Wilcoxon–Mann–Whitney test (A, B, C). ND_Fem: Female offspring born to lean mothers n = 8, F_HFD: Female offspring born to obese mothers n = 7, M_ND male offspring born to lean mothers n = 7, M_HFD male offspring born to obese mothers n = 9. Scale bar: 100µm.

# Supplementary data

## Fig. S3

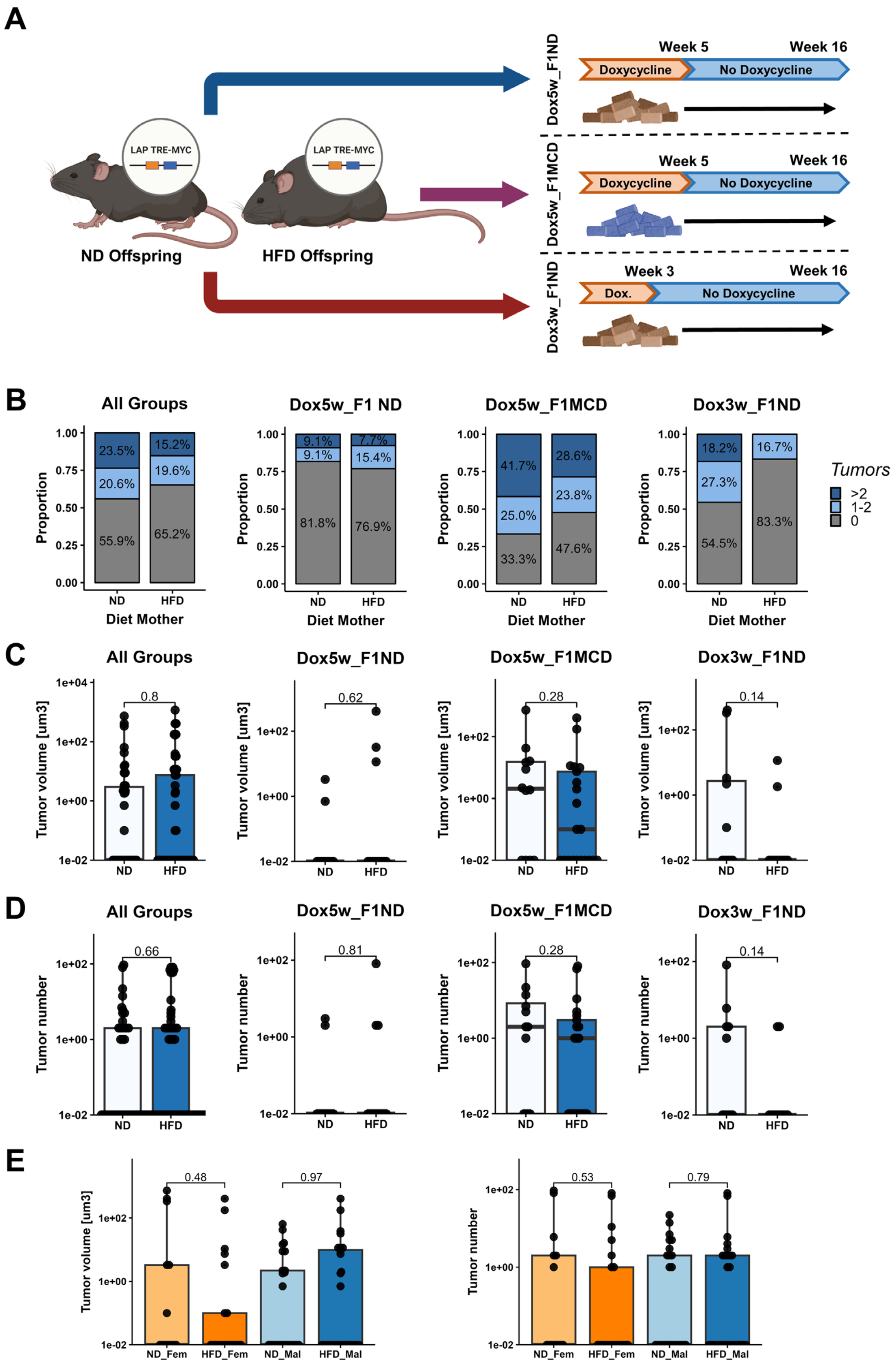

**Fig. S3. Maternal obesity does not influence the risk to develop HCC in a short-term transgenic HCC model** (**A**) HCC carcinogenesis in double transgenic offspring (LAP-tTA x TRE-MYC) of obese or lean mothers was induced through removal of doxycycline. Offspring were assigned to one of the following groups: Stop of doxycycline at 5 weeks with feeding of normal diet (ND) (Dox5w_F1ND), stop of doxycycline at 5 weeks with feeding of methionine choline deficient diet (MCD) (Dox5w_F1MCD), or stop of doxycycline at 3 weeks of age with feeding of a ND (Dox3w_F1ND) All animals were sacrificed at 16 weeks. (**B**) The proportion of animals that developed 0, 1-2 or more than 2 tumors for all groups on the left or per group on the right. (**C**) The total number of tumors per animal and (D) the total tumor volume per animal. (**E**) The total tumor volume or number differentiated by diet of the mother and sex of the offspring. Data presented as median ± IQR, one dot represents one animal, level of significance of p=0.05. Statistical analysis was performed by Wilcoxon–Mann–Whitney test (C, D, E). Median with interquartile ranges. Dox5w_F1ND n=23 (ND=11 and HFD=12), Dox5w_F1MCD n=33 (ND=12, HFD=21, Dox3w_F1ND n=24 (ND=11, HFD=13).

# Fig. S4

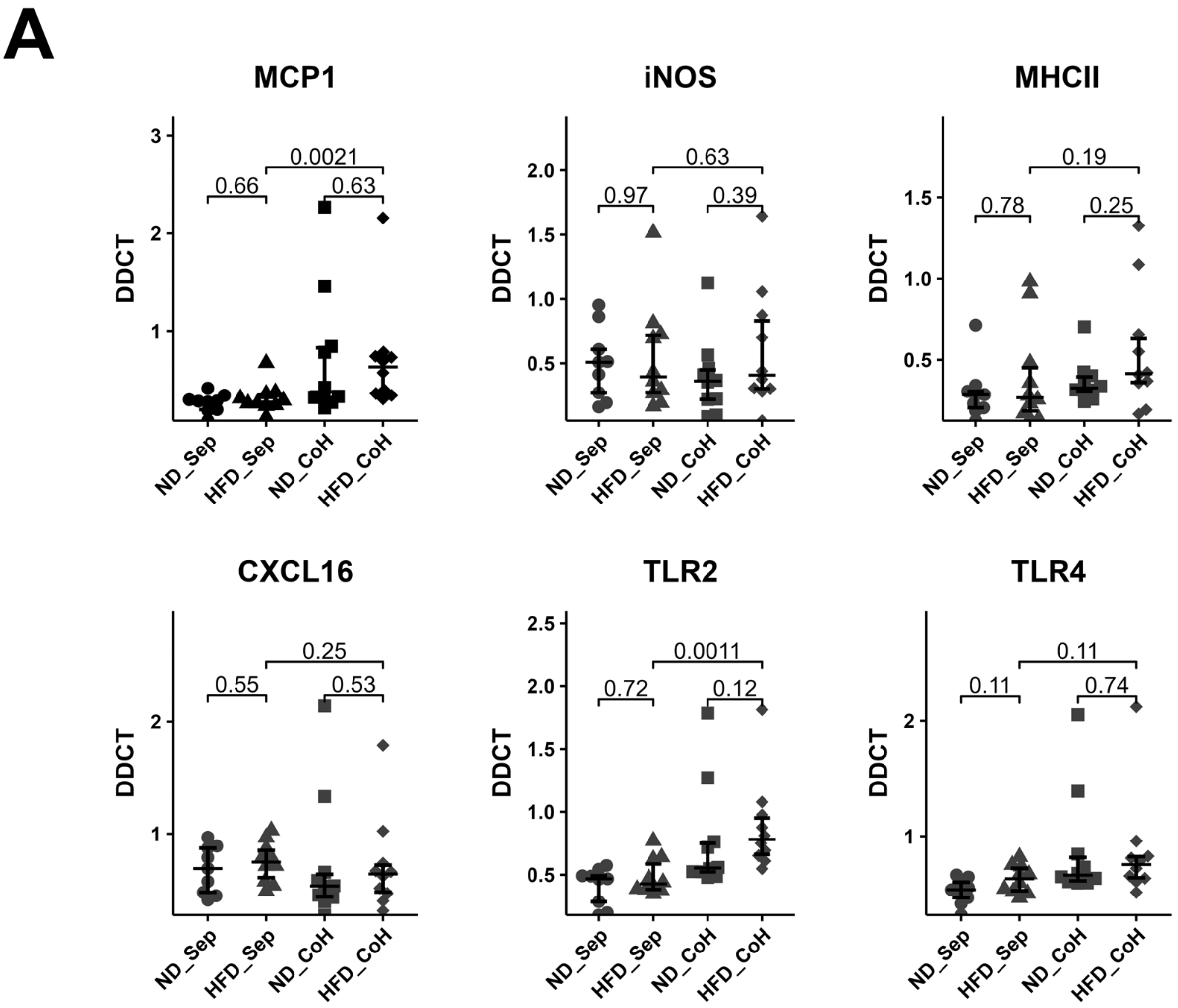


**Fig. S4. Expression of inflammatory markers or Toll-like receptor family members in DEN treated offspring** Offspring of obese and lean mothers were either separately housed (ND_Sep and HFD_Sep) or co-housed (ND_CoH and HFD_CoH) after weaning. (**A**) Real-time PCR expression analysis of inflammatory markers such as Monocyte Chemoattractant Protein-1 (MCP-1), inducible Nitric Oxide Synthase (iNOS), Class II Major Histocompatibility Complex (MHC II) molecules, and chemokine CXCL16. Expression of Toll-like Receptor (TLR) family member 2 and 4. Data presented as median ± IQR, one dot represents one animal, level of significance of p=0.05. Statistical analysis was performed by Wilcoxon–Mann–Whitney test. ND_Sep: Female offspring born to lean mothers housed seperately n = 10, HFD_Sep: Female offspring born to obese mothers housed separately n = 10, ND_CoH female offspring born to lean mothers co-housed with offspring of obese mothers n = 10, HFD_CoH female offspring born to obese mothers cohoused with offspring of lean mothers n = 10

# Supplementary references

[1] Felsher DW, Bishop JM. Reversible Tumorigenesis by MYC in Hematopoietic Lineages. Mol Cell 1999;4:199–207. https://doi.org/10.1016/S1097-2765(00)80367-6.

[2] Shachaf CM, Kopelman AM, Arvanitis C, Karlsson Å, Beer S, Mandl S, et al. MYC inactivation uncovers pluripotent differentiation and tumour dormancy in hepatocellular cancer. Nature 2004;431:1112–7. https://doi.org/10.1038/nature03043.

[3] Jackson DN, Panopoulos M, Neumann WL, Turner K, Cantarel BL, Thompson-Snipes L, et al. Mitochondrial dysfunction during loss of prohibitin 1 triggers Paneth cell defects and ileitis. Gut 2020;69:1928–38. https://doi.org/10.1136/gutjnl-2019-319523.

[4] Clevers HC, Bevins CL. Paneth cells: maestros of the small intestinal crypts. Annu Rev Physiol 2013;75:289–311. https://doi.org/10.1146/annurev-physiol-030212-183744.

[5] Johnson LA, Banerji S, Lawrance W, Gileadi U, Prota G, Holder KA, et al. Dendritic cells enter lymph vessels by hyaluronan-mediated docking to the endothelial receptor LYVE-1. Nat Immunol 2017;18:762–70. https://doi.org/10.1038/ni.3750.

[6] Mouries J, Brescia P, Silvestri A, Spadoni I, Sorribas M, Wiest R, et al. Microbiota-driven gut vascular barrier disruption is a prerequisite for non-alcoholic steatohepatitis development. J Hepatol 2019;71:1216–28. https://doi.org/10.1016/j.jhep.2019.08.005.

[7] Bankhead P, Loughrey MB, Fernández JA, Dombrowski Y, McArt DG, Dunne PD, et al. QuPath: Open source software for digital pathology image analysis. Sci Rep 2017;7:16878. https://doi.org/10.1038/s41598-017-17204-5.
